# Advancing battery failure diagnosis by knowledge-augmented large language models

**DOI:** 10.1093/nsr/nwag348

**Published:** 2026-06-08

**Authors:** Xin Zhang, Jingling Yuan, Lin Li, Zhaohui Deng, Jinqiao Du, Wen Luo, Liqiang Mai

**Affiliations:** Hubei Key Laboratory of Transport Internet of Things, School of Computer Science and Artificial Intelligence, Wuhan University of Technology, Wuhan 430070, China; Hubei Key Laboratory of Transport Internet of Things, School of Computer Science and Artificial Intelligence, Wuhan University of Technology, Wuhan 430070, China; Hubei Key Laboratory of Transport Internet of Things, School of Computer Science and Artificial Intelligence, Wuhan University of Technology, Wuhan 430070, China; State Key Laboratory of Advanced Technology for Materials Synthesis and Processing, Wuhan University of Technology, Wuhan 430070, China; Guangdong Provincial Key Laboratory of Source-Grid-Load-Storage Interactive Collaborative Technology, Shenzhen Power Supply Co., Ltd., Shenzhen 518000, China; State Key Laboratory of Advanced Technology for Materials Synthesis and Processing, Wuhan University of Technology, Wuhan 430070, China; Department of Physics Science and Technology, School of Physics and Mechanics, Wuhan University of Technology, Wuhan 430070, China; State Key Laboratory of Advanced Technology for Materials Synthesis and Processing, Wuhan University of Technology, Wuhan 430070, China

**Keywords:** battery failure diagnosis, large language model, knowledge-augmented generation, AI for battery

## Abstract

Battery failure diagnosis is crucial for ensuring the safety and reliability of energy storage systems. However, existing approaches from electrochemical modeling to deep learning often face limitations, including heavy reliance on extensive training data, poor generalization, and interpretability issues. To address these challenges, we propose BattFailScholar, a knowledge-augmented large language model (LLM) framework for battery failure diagnosis. Our approach first constructs a case-level battery failure knowledge graph encompassing material properties, multi-source signals, and failure pathways. A knowledge-augmented generation method is then developed to enhance LLM diagnostic reasoning with failure feature-aware retrieval and optimization algorithms. Experimental results demonstrate that BattFailScholar achieves a 19.7% performance improvement in LLM-based diagnosis, with enhanced capability in alleviating long-tail problems and failure risk assessment. Moreover, the system achieves 86.2% accuracy in identifying potential failure mechanisms or causes, demonstrating strong potential for discovering failure chains and providing practical, reliable diagnostic support for battery research and development.

## Teaser

This work proposes BattFailScholar, a novel knowledge-augmented large language model framework that learns from extensive battery failure cases, offering a new solution for accurate, evidence-based battery failure diagnosis.

## INTRODUCTION

**Figure 1.**
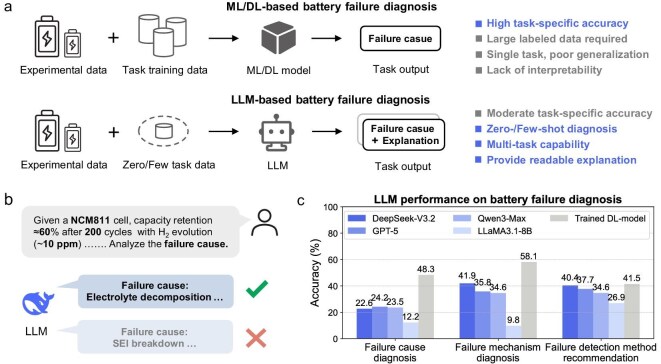
Large language model (LLM)-based battery failure diagnosis: overview and challenges. (a) Comparison of advantages and limitations between machine learning (ML)/deep learning (DL) and LLM methods for battery failure diagnosis. (b) Example of LLM-based failure cause diagnosis, where experimental data are provided to an LLM in text form to generate diagnostic results. (c) Performance of current mainstream LLMs on failure diagnosis tasks, showing limited accuracy for direct application.

Battery failure diagnosis serves as a cornerstone for maintaining the operational integrity, safety, and durability of modern energy storage systems. By identifying failure forms, mechanisms, and causes, it enables early warning of performance degradation, guides material and system design, and supports the development of safer and longer-lasting batteries [1–5]. Across diverse research and engineering scenarios, failure diagnosis has been widely applied to uncover degradation pathways, monitor multi-physical states (for example, temperature, voltage, and impedance), and inform the management of thermal, electrical, and structural stability in practical battery systems [6–8]. These advances have substantially deepened our understanding of failure phenomena across multiple scales, from electrochemical degradation at interfaces to system-level safety management. However, existing diagnostic paradigms [9–11], ranging from empirical experience to mechanistic modeling and data-driven learning approaches, including machine learning (ML) and deep learning (DL), still face several fundamental challenges, as depicted in Fig. 1a. These methods typically require large amounts of well-labeled failure data for training, which are often scarce and expensive to obtain [10,12]. More importantly, their high accuracy on specific tasks, achieved through data fitting, often comes at the cost of poor generalization across different battery chemistries or failure types, while still relying heavily on domain expertise for feature engineering [13–15]. Furthermore, these methods rarely provide causal explanations and diagnostic evidence [9], further limiting their practical value.

The emergence of large language models (LLMs) has transformed the landscape of scientific analysis and knowledge discovery. By leveraging vast pre-trained data and powerful reasoning abilities, LLMs have demonstrated remarkable performance in tasks such as complex question reasoning [16], and decision making [17]. Recent studies have further explored their role in diverse applications including materials design [18–20], automated synthesis development [21], catalysis pathway recommendation [22], and biological evidence generation [23]. For battery failure diagnosis, LLMs offer distinct advantages here that address key limitations of conventional approaches, specifically as demonstrated in Fig. 1a. First, through their zero- and few-shot learning capabilities, they perform diagnosis with minimal labeled data, thereby directly lowering the data barrier [24]. Second, their strong semantic understanding and knowledge association capabilities facilitate multi-task diagnosis across diverse battery chemistries and failure types [25]. Additionally, LLMs provide explicit diagnostic rationales, connecting observed signals to underlying degradation causes through human-interpretable reasoning [26]. Therefore, in contrast to ML and DL approaches, we aim to propose an LLM-based approach for battery failure diagnosis, as demonstrated in Fig. 1b, which illustrates a concrete LLM-based failure cause identification example. This approach introduces a new paradigm for widely applicable and reliable diagnostic reasoning in battery systems.

**Figure 2.**
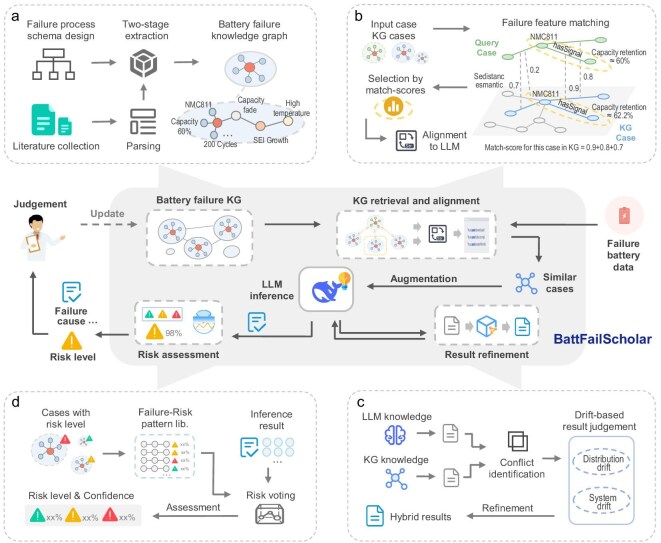
The framework of the proposed BattFailScholar. (a) Construction of a case-level battery failure knowledge graph (BF-KG) that provides a structured failure knowledge base encompassing battery materials, multi-source signals, and failure evolution pathways. (b) Failure-feature-aware case retrieval algorithm that identifies and retrieves the most diagnostically relevant reference cases from the knowledge graph to augment LLM reasoning. (c) Drift-aware result correction mechanism that detects conflicts between LLM knowledge and retrieved cases, dynamically adjusting outputs to mitigate long-tail issues and improve diagnostic accuracy for low-frequency failure types. (d) Pattern-based voting mechanism for quantitative risk assessment, which integrates diagnostic information from multiple cases to compute confidence scores for different risk levels, enabling reliable risk estimation.

Despite their impressive capabilities, current LLMs still face critical limitations when applied to battery failure diagnosis, as shown in Fig. 1c where experimental results on multiple failure diagnostic tasks reveal that current LLMs achieve limited accuracy and are difficult to deploy directly. This issue primarily stems from their dependence on limited pre-trained knowledge, which often leads to inaccurate reasoning [27] when handling complex domain-specific concepts [28]. These challenges align with findings from other scientific domains, where studies have documented similar issues in materials discovery [18], biomedical reasoning [23], and chemical reaction prediction [22], with models frequently generating plausible yet scientifically incorrect outputs.

To enhance the factual accuracy and scientific grounding of LLMs, recent studies have proposed strategies, including domain-specific fine-tuning (FT) [29] and retrieval-augmented generation (RAG) [30]. While FT can improve reasoning depth, they require extensive data and lack flexibility when scientific knowledge rapidly evolves [31]. RAG addresses these limitations by retrieving external data during generation. This adaptability makes it particularly suitable for knowledge-intensive and rapidly evolving domains, such as battery failure diagnosis. Building on this foundation, knowledge-augmented generation (KAG) further incorporates structured knowledge resources such as knowledge graphs [32], supporting domain-grounded reasoning. KAG has shown promising results across scientific fields [18,22,23]. However, extending KAG to battery failure diagnosis presents critical challenges, including the lack of structured failure knowledge, complex multi-source signals, diverse failure types, and the need for reliable risk assessment.

In this study, we propose BattFailScholar, a knowledge-augmented LLM framework for accurate and reliable battery failure diagnosis. The framework begins by constructing a case-level knowledge graph from scientific literature, capturing material characteristics, multi-source signals, and failure chains. To effectively retrieve reference cases from a large case base with complex signals, a failure-feature-aware retrieval algorithm is proposed for the LLM. For long-tail low-frequency failure types, a drift-aware correction mechanism is designed to refine diagnostic results. Finally, a pattern-based voting algorithm is developed that outputs risk levels with confidence scores, enabling reliable risk assessment. Experimental results show that BattFailScholar significantly improves the accuracy of LLM diagnostics, surpassing trained deep learning models, while effectively balancing low-frequency failure types and providing more reliable risk levels. In a failure chain completion study, BattFailScholar effectively demonstrated its capability to uncover unrecognized failure pathways. Overall, this approach provides a new pathway for battery failure diagnosis with lower usage barriers, competitive accuracy, and broader applicability, supporting battery researchers in identifying complex failures across diverse battery scenarios.

## RESULTS AND DISCUSSION

### Framework overview

BattFailScholar is a knowledge-augmented LLM framework designed to advance battery failure diagnosis from conventional data-driven approaches toward knowledge-driven paradigm. By augmenting general-purpose LLMs with systematically curated prior failure cases from the literature, the framework equips these models with domain-specific expertise, enabling diagnostic capabilities that transcend the limitations of traditional methods. As illustrated in Fig. 2, the framework consists of four core modules. These modules work collaboratively to form a complete diagnostic workflow: knowledge graph construction establishes the knowledge foundation, failure-feature-aware retrieval enables precise case matching, drift-aware correction optimizes diagnosis for low-frequency failures, and pattern-based voting outputs quantitative risk assessments. The diagnostic results are then provided to users or downstream systems for informed decision-making or automated response, and can subsequently be incorporated back into the knowledge graph for continuous expansion.

To address the lack of a structured battery failure knowledge base, a case-level BF-KG was constructed through literature mining, as illustrated in Fig. 2a, inspired by the case-based reasoning (CBR) principle of organizing knowledge around experiential cases [33]. A key innovation lies in the KG architecture. Built at the case level, our framework comprehensively captures material properties and attributes, multi-source signals (electrical, thermal, gas, and mechanical signatures) and failure chains. To hierarchically represent failure evolution, specialized node types enable dynamic modeling of degradation pathways from initial conditions to final failure modes. For knowledge extraction, a two-stage LLM-enabled open information extraction method is employed, moving beyond rigid predefined schemas [34] by leveraging semantic understanding [35] to capture both predefined and newly discovered concepts. More details on BF-KG are provided in Sections S1 of the supplementary information (SI).

For effective knowledge retrieval to augment LLM-based diagnosis, a failure-feature-aware algorithm is introduced. It first pre-filters the knowledge graph to retain cases with matching failure forms. Fine-grained semantic matching then quantifies pairwise similarities between query and candidate case triplets via embedding representations, as illustrated in Fig. 2b. Overall similarity is aggregated from optimal triplet matches, retrieving the top-k most relevant cases for diagnosis. Further details are in Section S2.1. Unlike conventional graph retrieval methods [36], our algorithm enhances retrieval precision by incorporating failure-feature awareness at the case level, addressing the key challenge where similar external manifestations may stem from different underlying factors. With the retrieved references and input data prepared, they are jointly converted into a format most comprehensible to the LLM to fully leverage its capabilities. The system then automatically loads task-specific instructions and candidate labels for reasoning. The prompt is available in Section S2.2.

Despite the retrieved cases enhancing LLM reasoning, the diversity of failure types introduces a long-tail challenge where low-frequency failures remain difficult to diagnose reliably. These types have scarce samples in the case base, leading to poorly matched retrieved references that may interfere with LLM reasoning. This creates conflicts between the model’s internal knowledge and retrieved external knowledge [37], leading KAG-based diagnosis to exhibit a correct-to-incorrect shift compared to the base LLM and resulting in misclassification of true failure types. To address this, a drift-aware correction mechanism is innovatively proposed. As illustrated in Fig. 2c, it monitors two key indicators, label distribution shifts and chemical system shifts, to assess knowledge conflict (for more details see Section S3.1). Based on the detected drift degree, the system dynamically selects either the original LLM reasoning or KAG-enhanced outputs, enabling more balanced diagnostic performance across diverse failure types.

Beyond diagnosing failure causes and mechanisms, reliable failure risk assessment is equally critical for the safety and operational reliability of battery management systems. However, existing direct LLM-based methods, despite certain reasoning capabilities, often produce overconfident outputs [38], limiting their direct applicability in practical scenarios. To address this, as illustrated in Fig. 2d, a pattern matching and ensemble voting-based risk assessment method is proposed. A ‘Failure-Risk’ pattern library covering typical failure modes is first constructed as a prior knowledge base. The Top-N predicted failure information is then matched with cases in the library, and confidence levels for each risk category (for example, high, medium, and low) are calculated through multi-case ensemble voting. More methods are detailed in Section S3.2. This method no longer relies on judgments from single cases; instead, it integrates fault diagnosis information from multiple cases to effectively mitigate the impact of individual case bias, thereby ensuring the credibility of the assessment results.

**Figure 3.**
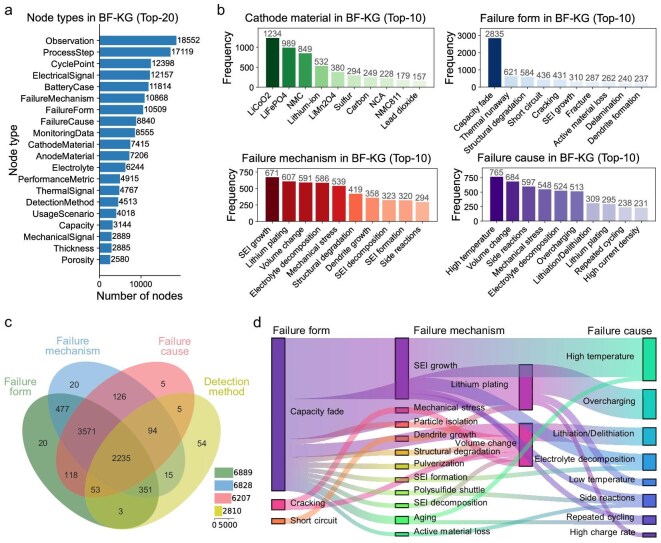
Construction results and statistics of the BF-KG. (a) Node statistics in the graph, including material properties, multi-source signals, failure information, and structural nodes. (b) Entity statistics under partial core type nodes, covering cathode/anode materials and key failure type nodes. (c) Statistics on the occurrence of various failure information in the literature used for graph construction. (d) Sankey diagram of partial high-frequency failure chains under common failure types in BF-KG, revealing the diagnostic complexity of battery failures, where similar symptoms stem from distinct pathways.

### Statistics of the constructed BF-KG

Extensive battery failure knowledge is embedded in scientific literature but remains unstructured and scattered. To systematically acquire this knowledge, 10 170 academic publications on lithium-ion battery failure were collected from Google Scholar through automated queries, forming the comprehensive corpus. Based on the proposed extraction approach, and after a series of data cleaning steps, BF-KG was successfully constructed from 7309 publications.

The constructed BF-KG comprises 181 694 nodes and 177 520 edges, including 11 814 failure cases that form a substantial structured repository of battery failure knowledge. As shown in Fig. 3a, the distribution of the top 20 entity types reveals the BF-KG’s comprehensive coverage, with core failure-related nodes, including Failure Form (10 480), Failure Mechanism (10 828), and Failure Cause (8829) constituting the backbone of the failure knowledge structure. Additionally, BF-KG incorporates extensive material properties and multi-physics signals, providing a rich contextual basis for failure analysis. More detailed nodes can be found in Section S1.4. The distribution of key entity types is further provided in Fig. 3b. The statistics for cathode/anode materials, along with failure forms, mechanisms, causes, and detection methods, reveal the research community’s focus areas and knowledge distribution patterns.

The coverage of four critical failure data elements across the 7309 publications was also examined, with statistical results shown in Fig. 3c. The findings indicate that while complete failure chains are not extracted from every publication, the majority contain information on failure forms, mechanisms, or causes. Additionally, high-frequency failure chains across common failure types in the BF-KG were statistically analyzed and visualized as a Sankey diagram in Fig. 3d. These partial chains clearly illustrate the relationships between failure forms, mechanisms, and causes, with capacity decay emerging as the predominant failure form. The diverse mechanisms and causes underlying this single failure form highlight the diagnostic complexity of battery failures, where similar symptoms stem from distinct pathways, necessitating the development of advanced diagnostic approaches.

**Figure 4.**
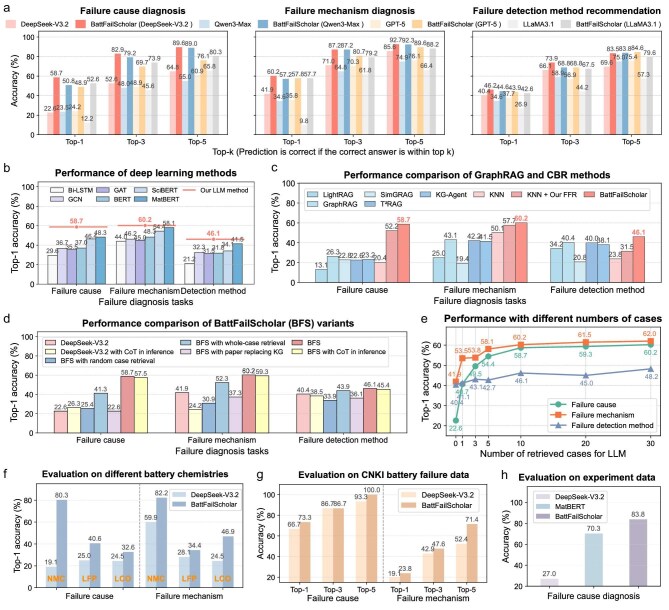
Performance evaluation of the BattFailScholar framework. (a) Performance comparison between general LLMs and BattFailScholar-enhanced LLMs on battery failure diagnosis. (b) Performance of common DL methods, demonstrating the potential of LLMs-based methods. (c) Comparative experiments with mainstream GraphRAG and case-based reasoning methods. (d) Ablation study of BattFailScholar: validating the advantages of the proposed graph retrieval algorithm and graph representation. (e) Effect of retrieved case quantity on diagnostic performance, exhibiting a positive correlation. (f) Diagnostic performance across different battery chemistries, validating the method’s adaptability to diverse chemical systems. (g) Evaluation on cross-source data to verify generalization capability beyond the original source. (h) Performance on real experimental datasets, demonstrating practical applicability in real-world scenarios.

### Performance evaluation of the proposed LLM-based diagnosis approach

To enable quantitative evaluation, a battery failure diagnosis benchmark was constructed from well-structured cases in the knowledge graph. It comprises two tasks: failure cause and mechanism diagnosis (FCFM) and failure detection method recommendation (FDM). For FCFM, test cases were selected to simultaneously contain Failure Form, Failure Mechanism, and Failure Cause nodes, with one instance per node type to ensure clear causal relationships. To maintain a representative label distribution, cases containing at least one of the top 30 most frequent labels per category were retained, resulting in 327 valid cases as a test set. For FDM, which recommends appropriate detection methods for validating suspected failure modes, a test set of 260 cases was constructed analogously. For each task, the remaining BF-KG cases (excluding their respective test set) form the retrieval library for case-based reasoning. To address label variations from LLM-based extraction, equivalent expressions were unified through semantic analysis and expert consultation. More dataset construction details are available in Section S4.1.

For model evaluation, several representative LLMs were selected, including the latest mainstream LLMs (DeepSeek-V3.2 [39], Qwen3-Max [40], and GPT-5 [41]) and the lightweight open-source LLaMA3.1-8B [42] suitable for local deployment. As a plug-and-play framework, BattFailScholar was integrated with each base LLM and evaluated against baseline methods to demonstrate its diagnostic performance improvement.

As for the performance evaluation metric, Accuracy (Top-1, Top-3, and Top-5) was adopted. Top-1 requires exact match with ground truth, reflecting precise diagnostic capability, while Top-3 and Top-5 help narrow down potential failure types under limited data by considering a prediction correct if the ground truth appears among the top-k recommendations. More experimental details are provided in Section S4.2.

Based on this evaluation setup, comprehensive experiments were conducted comparing the proposed BattFailScholar framework with selected baseline LLMs. As shown in Fig. 4a, when augmented with our BattFailScholar framework, all evaluated LLMs exhibit substantial performance improvements. The knowledge augmentation approach achieves average accuracy gains of 27.3%, 19.7%, and 12.0% across the three diagnostic tasks respectively, demonstrating the effectiveness of BatFailScholar. A detailed per-failure type diagnostic performance is provided in Section S4.3.

Among the evaluated models, DeepSeek-V3.2 achieves the most competitive performance, with Top-3 and Top-5 accuracy exceeding 80% and even reaching 92.7%, respectively. Such performance levels can substantially assist researchers in narrowing down potential failure types. Nevertheless, despite these improvements, Top-1 accuracy only reaches 60%, potentially due to limited experimental data in the input and the inherent diagnostic challenge where similar failure manifestations may stem from multiple underlying causes or mechanisms.

To establish comprehensive comparisons with conventional deep learning approaches, comparative experiments were conducted with several representative methods, such as Bi-LSTM [43], BERT variants [44,45], and GNN-based models [46,47], as summarized in Fig. 4b. In contrast to the BattFailScholar that leverages a limited set of relevant cases, these methods used the entire case base as supervised training data. More details are provided in Section S4.4. Results demonstrate that our LLM-enhanced approach achieves comparable or even superior performance to these methods trained on the full case base, underscoring the promise of LLMs for accurate battery failure diagnosis. Additionally, it is worth noting that when case knowledge is scarce (that is, zero-shot scenarios), these deep learning methods become ineffective, whereas LLMs can leverage their knowledge to achieve baseline diagnosis, as illustrated in Fig. 4a.

Additionally, comparisons were conducted with mainstream GraphRAG [36] and CBR-related methods (complete introduction is provided in Section S4.5), with results shown in Fig. 4c. Most GraphRAG-based methods proved inadequate, often showing no improvement or even performance degradation due to noise, as they perform entity-level multi-hop retrieval without integrated case-level feature consideration. KNN, as a representative CBR method [48], achieved slight improvement but remained limited due to unfiltered noise features. After integrating our failure feature-aware retrieval (FFR), performance improved substantially, yet still lagged behind LLM-based results, highlighting the necessity of LLM reasoning with the same case data.

Further ablation studies evaluated component contributions in BattFailScholar using several variants (see Section S4.6), with results in Fig. 4d. First, chain of thought prompting [49] showed no improvement, indicating reasoning is not the bottleneck. Second, replacing our retrieval with random case retrieval or node-undifferentiated retrieval degraded performance, validating our failure-aware design. Finally, using paper excerpts instead of structured cases substantially degraded performance, as lengthy, low-density contexts likely overwhelmed the LLM. This confirms the superiority of structured knowledge graphs over raw text.

To validate the effectiveness of providing reference cases to LLMs, parameter sensitivity experiments were conducted on the number of retrieved cases, as shown in Fig. 4e. Results show a positive correlation between case quantity and LLM performance, confirming the effectiveness of retrieved cases in enhancing diagnosis. Performance plateaus at around 10 cases, likely due to context length limits and less relevant cases. Based on this, the default case number was set to 10 throughout experiments. The relatively smaller performance gain in detection method recommendation can be attributed to the limited reference cases, as shown in Fig. 3c.

Moreover, additional evaluations were conducted, including clustering analysis of BF-KG (Section S4.9), case study (Section S4.10), interpretability and reliability evaluation (Section S4.11), efficiency evaluation (Section S4.12), and a diagnostic platform application example (Section S6.1), with limitations discussed in Section S6.2.

**Figure 5.**
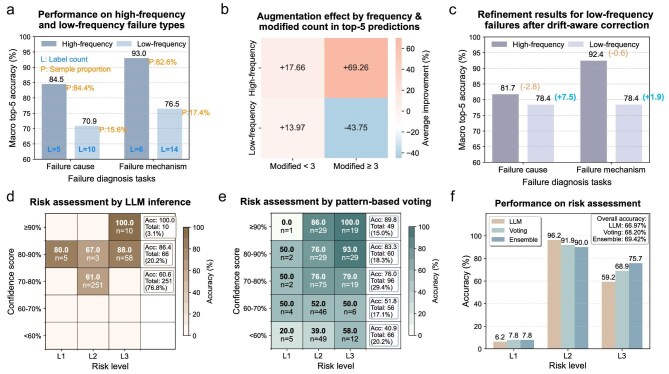
Long-tail refinement results for failure types and risk assessment performance. (a) Long-tail problem in current diagnostic results: high vs. low frequency. (b) After augmentation, low-frequency failure data with substantial prediction shifts tend to become incorrect rather than improved, due to noise introduced by scarce reference cases. (c) Results optimized by the proposed drift-aware correction mechanism, showing improved accuracy for low-frequency failure types. (d) Risk assessment performance and confidence distribution of LLM, exhibiting uniform and consistently high confidence scores. (e) Performance of the proposed pattern-based voting method, demonstrating reasonable confidence distribution where higher confidence corresponds to better accuracy. (f) Comparison of LLM-based, voting-based, and hybrid methods, validating the advantage of the proposed approach, particularly for high-risk scenarios.

### Generalization evaluation on different failure diagnostic systems and domains

To further evaluate the generalization capability of the proposed method, performance comparisons across different battery chemistries were first analyzed. The three most abundant types, NMC (nickel manganese cobalt), LFP (lithium iron phosphate), and LCO (lithium cobalt oxide), were selected for evaluation, with results shown in Fig. 4f. The most significant improvement was observed on NMC, where Top-1 accuracy reached 80%, demonstrating that sufficient case support enables highly reliable diagnostic performance, while substantial improvements were also achieved on LCO and LFP. This validates that the proposed method can effectively support failure diagnosis across different battery chemistries.

Furthermore, since the test set and case base were constructed from the same source, they share a certain homogeneity. To further validate generalization of BattFailScholar, an additional test set was constructed by collecting relevant failure data from another source (CNKI). The originally constructed case base was still used for retrieval augmentation, with results shown in Fig. 4g. Performance improvements were still achieved even with failure data from different sources, validating the cross-source generalization capability of the proposed approach. Further details are available in Section S4.7.

Additionally, the practical applicability of BattFailScholar was further validated on real laboratory data using thermal runaway battery failure datasets [50], where the task is to diagnose the causes leading to thermal runaway based on actual experimental records. As shown in Fig. 4h, by retrieving the most relevant data as references, the proposed LLM-based approach achieves comparable or even superior performance to trained deep learning methods. These results demonstrate the method’s ability to generalize from literature-derived data to real experimental data, confirming its practical utility for battery failure diagnosis tasks. More details are provided in Section S4.8.

### Long-tail problem of failure types and reliable risk assessment

Due to the wide variety of failure types and the inherent limitations of case base data, long-tail problems inevitably arise in battery failure diagnosis. Statistical analysis was conducted on the augmented results, where failure causes and mechanisms were categorized into high-frequency and low-frequency types based on their occurrence counts in the case base (types with fewer than 100 occurrences were defined as low-frequency). As shown in Fig. 5a, low-frequency failure types significantly outnumber high-frequency types, yet their data proportion and average diagnostic accuracy are substantially lower, revealing a pronounced long-tail challenge (Section S4.13 for detailed analysis of failure type long-tail distribution). This leads to insufficient diagnostic reliability for low-frequency types, making it difficult to effectively identify diverse failure scenarios in practical applications.

Further analysis of Top-5 predictions (Fig. 5b) reveals that for low-frequency types, substantial prediction changes after augmentation often lead to misclassification due to noisy retrieved references. In contrast, high-frequency types benefit from sufficient relevant cases, enabling effective error correction. However, since low-frequency types cannot be identified in advance, direct correction based on type frequency is infeasible. To address this, a drift-aware correction mechanism was proposed. When substantial label changes co-occur with significant chemical system shifts, such discrepancies indicate potential misclassification of low-frequency types caused by noisy retrieved cases, triggering reversion to base LLM predictions. As shown in Fig. 5c, this mechanism improves diagnostic accuracy for low-frequency types with minimal impact on high-frequency types. Although the improvements may appear modest (about 7.5% and 1.9%), achieving such gains is particularly challenging given the limited data and diverse nature of low-frequency failure types, enabling more balanced identification across diverse failure categories. Further details on the prediction change analysis and correction effectiveness are provided in Section S4.13.

In addition to failure diagnosis, reliable risk assessment is also critical. For comparative evaluation, the test set was annotated with risk levels (L1: low-risk, L2: medium-risk, and L3: high-risk) to form a risk assessment benchmark (as detailed in Section S4.14). Given experimental data and identified failure causes/mechanisms, the goal is to predict risk levels with confidence scores. Conventional LLM-based methods tend to produce high but uniform confidence scores (Fig. 5d), all exceeding 70% and mostly concentrated in a narrow 70% to 80% range, offering limited practical value.

To address this, we proposed a pattern-based voting method. It first statistically analyzes correlations between failure patterns and risks to construct a failure-risk pattern library (Section S4.14 provides detailed library statistics and representative patterns). During assessment, Top-5 diagnostic results are combined with this library for voting, yielding confidence scores for each risk level along with supporting evidence. As shown in Fig. 5e, this approach produces a more reasonable confidence distribution where higher scores correlate with better accuracy. Comparative results (Fig. 5f) show significantly improved performance for high-risk types. Notably, LLM-based methods achieve relatively good accuracy on high-confidence L3 predictions, demonstrating some capability in identifying high-risk scenarios. These high-confidence predictions were therefore integrated with voting results to combine strengths. As shown in Fig. 5f, this hybrid integration improves accuracy for high-risk cases while maintaining performance for other categories, offering a optional solution.

**Figure 6.**
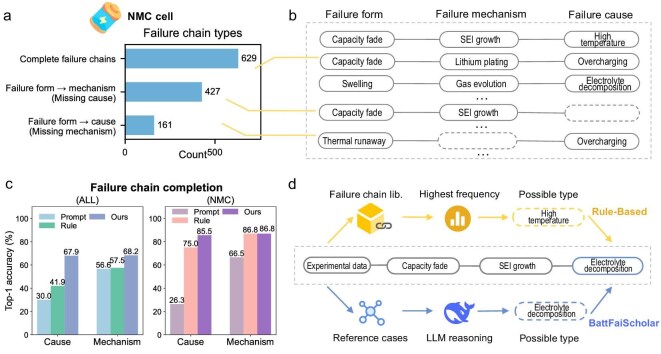
Failure chains of NMC batteries in BF-KG and evaluation of failure cause/mechanism discovery. (a) Statistics of failure chains in NMC battery cases within BF-KG, showing prevalent absence of failure mechanisms and causes. (b) Representative high-frequency failure chains for NMC batteries, including both complete and incomplete patterns. (c) Performance comparison of prompt-based, rule-based, and BattFailScholar approaches in discovering failure mechanisms and causes. The rule-based method selects highest-frequency matches from the chain database, while BattFailScholar employs case retrieval for LLM reasoning. (d) Example comparison between rule-based and BattFailScholar approaches for failure cause identification.

### Discussion

To validate the practical application of BattFailScholar in real-world battery failure scenarios, a focused study was conducted on NMC batteries. The failure chains of NMC cathode cases in the knowledge graph were first analyzed. The statistics in Fig. 6a reveal incomplete failure chains across many cases, with substantial numbers missing either failure causes or mechanisms. Figure 6b illustrates representative chain patterns, with more examples available in Section S5.1.

Inferring these missing elements provides significant value for battery research and development, offering crucial insights for designing more robust systems and enabling proactive failure prevention. To evaluate this capability, NMC cathode samples were systematically masked by removing either failure mechanisms or causes, requiring the model to infer missing elements from available information. Comprehensive results on the full evaluation dataset are shown in Fig. 6c. Three approaches were compared: direct LLM prompting; a rule-based method matching remaining chain elements against the NMC failure chain library and selecting the most frequent occurrence; and BattFailScholar, which retrieves relevant cases from BF-KG to augment LLM reasoning (Section S5.2).

Results reveal the limitations of relying solely on statistical frequency in failure chain completion. As shown in Fig. 6d, while the frequency-based approach improves accuracy over direct LLM inference, its deterministic nature cannot adequately represent multifactorial degradation pathways in real battery systems. In contrast, BattFailScholar effectively integrates experimental data with relevant case references, achieving overall improvements of 26.0% and 10.7% over the rule-based method on the full evaluation dataset, while reaching accuracies of 85.5% and 86.8% on the NMC subset for failure cause and mechanism completion, respectively. This performance level demonstrates practical utility for real-world applications, enabling automated failure chain construction in battery diagnostic systems. Beyond these quantitative gains, our method offers a promising paradigm for scientific reasoning in battery failure diagnosis. By integrating structured domain knowledge with flexible neural reasoning, it provides a practical approach to handling the complexity and diversity of real-world battery degradation, delivering interpretable and evidence-based diagnostic support for materials scientists.

## CONCLUSIONS

We present BattFailScholar, a knowledge-augmented LLM framework for battery failure diagnosis. The system is built upon a case-level knowledge graph of 11 814 battery failure cases constructed from scientific literature, enabling evidence-based diagnosis through failure-feature-aware retrieval and optimization algorithms. Through systematic evaluation, BattFailScholar achieves a 19.7% average performance improvement across multiple diagnostic tasks compared to leading LLMs. The framework also effectively alleviates long-tail problems for low-frequency failure types and enables reliable risk assessment with interpretable confidence scores. Moreover, the system reaches 86.2% accuracy in identifying potential failure mechanisms or causes for NMC-based lithium batteries, demonstrating strong potential for discovering failure chains. The value of our approach lies in its ability to deliver precise diagnostics with verifiable evidence while maintaining broad applicability across different battery chemistries and failure scenarios. This combination of accuracy, reliability, and generalization makes BattFailScholar a practical and trustworthy solution for LLM-assisted battery safety management. Future work will incorporate image data as a multi-modal complement to address more diverse information sources, further enhancing intelligent battery failure diagnosis.

## Supplementary Material

nwag348_Supplementary_data

## Data Availability

The proposed battery failure knowledge graph and the implementation code of BattFailScholar will be publicly available at https://github.com/xinzcode/BattFailScholar. Detailed documentation and data descriptions can be found in the supplementary data.
