# Supplementary material for: Advancing battery failure diagnosis by knowledge-augmented large language models
Source: Natl Sci Rev. 2026 Jun 8;13(14):nwag348. doi: 10.1093/nsr/nwag348 (PMC13382905; doi:10.1093/nsr/nwag348)
Supplement: nwag348_Supplementary_data [file nwag348_supplementary_data.pdf]

Supplementary Information

Advancing battery failure diagnosis by knowledge-augmented large language models

Xin Zhang[1], Jingling Yuan[1,*], Lin Li[1],
Zhaohui Deng[2], Jinqiao Du[4], Wen Luo[2,3,*], Liqiang Mai[2,*]

[1]Hubei Key Laboratory of Transport Internet of Things,
School of Computer Science and Artificial Intelligence,
Wuhan University of Technology, Wuhan, 430070, China.
[2]State Key Laboratory of Advanced Technology for Materials Synthesis and Processing,
Wuhan University of Technology, Wuhan, 430070, China.
[3]Department of Physics Science and Technology, School of Physics and Mechanics,
Wuhan University of Technology, Wuhan, 430070, China.
[4]Guangdong Provincial Key Laboratory of Source-Grid-Load-Storage Interactive
Collaborative Technology, Shenzhen Power Supply Co., Ltd., Shenzhen 518000, China.

***Corresponding author(s). E-mail(s):**
yjl@whut.edu.cn; luowen_1991@whut.edu.cn; mlq518@whut.edu.cn

Section 1. Battery Failure Knowledge Graph Construction

1.1 Literature Collection and Parsing

1.2 Failure-Process-Aware Schema Design

1.3 Text Extraction with LLMs

1.4 Knowledge Graph Data Statistics

Section 2. Knowledge-Augmented LLM for Battery Failure Diagnosis

2.1 Failure Feature-Aware Retrieval Algorithm

2.2 Task Prompt Build for Battery Failure Diagnosis

2.3 Illustrative Example of the Knowledge-Augmented LLM Diagnosis Process

Section 3. Long-Tail Problem of Failure Types and Reliable Risk Assessment

3.1 Drift-Aware Correction Mechanism

3.2 Pattern Matching-Based Voting Algorithm

Section 4. Evaluation Dataset and Experimental Details

4.1 Evaluation Dataset Construction

4.2 Experiment Details

4.3 Per-Failure Type Diagnostic Performance

4.4 Deep Learning Method Evaluation

4.5 Comparative Experiment

4.6 Ablation Experiment

4.7 Cross-Source Generalization Evaluation

4.8 Laboratory Data Evaluation

4.9 Failure Cluster Visualization of BF-KG

4.10 Case Study

4.11 Interpretability and Reliability Evaluation

4.12 Efficiency Evaluation

4.13 Long-Tail Problem Experiment

4.14 Risk Assessment Experiment

Section 5. Battery Failure Chain Completion

5.1 Battery Failure Chain Statistics

5.2 Task Prompt Build for Failure Chain Completion

Section 6. Framework Application and Limitations

6.1 Diagnostic Platform and Application Example

6.2 Limitations

References

Section 1. Battery Failure Knowledge Graph Construction

1.1 Literature Collection and Parsing

Currently, the field of battery research offers a wealth of public datasets, which can be broadly categorized as follows:

- **Long-term aging datasets [1, 2, 3]**: These record the macroscopic performance evolution of batteries during cycling, such as capacity fade and internal resistance growth.
- **Abuse test datasets [4, 5]**: These investigate the safety boundaries and thermal behavior of batteries under extreme conditions through thermal runaway experiments.
- **Operational condition datasets [6, 7, 8]**: These include charge-discharge curves and impedance data under various current rates and temperatures, used for performance evaluation.

These datasets document when and what performance changes occurred in batteries, yet remain collections of phenomena rather than systematic knowledge. They show what happened to the batteries but seldom explain why, such as the underlying failure mechanisms, causal relationships among patterns, or differences in degradation pathways across chemistries. However, a battery failure knowledge base that systematically integrates these phenomena and incorporates the underlying mechanisms remains lacking. Most of this knowledge is still scattered across diverse battery research literature. Therefore, we propose to extract the necessary knowledge on battery failure from this vast body of literature, with the aim of constructing a knowledge base that integrates multi-source information and reveals the failure evolution process. This will structure the fragmented failure knowledge, providing a mechanistic foundation for precise diagnosis and intelligent prediction. Specifically, the method is as follows:

1. We first performed a search on Google Scholar using the keyword "Li-ion battery failure" to identify relevant literature. Due to the limitation of accessible search results (capped at 100 pages), we introduced a filtering strategy: we conducted searches year by year to ensure comprehensive coverage. Using a Python script, we automated the retrieval of paper titles for each year from 2000 to 2025 and compiled the results.
2. Based on the retrieved titles, the Python script was used to obtain accurate Digital Object Identifiers (DOIs), which were then used to download the original PDFs of the papers.
3. Text parsing tools were employed to convert the PDFs into plain text files (.txt). Each file is named according to its DOI, and the content includes the title, abstract, and main body.

Through this process, we successfully assembled a corpus of 10,170 battery failure-related papers in text format.

1.2 Failure-Process-Aware Schema Design

In the safety and reliability analysis of energy storage batteries, the dynamic evolution characteristics of the failure process are crucial. Battery performance degradation during usage is often not caused by a single factor, but results from the intertwined evolution of multiple physical mechanisms such as material aging, operational anomalies, and thermal runaway. These processes exhibit features such as coupled electrical-thermal-gas-mechanical signals, temporal progression, and chain-reaction failures. However, existing knowledge graphs remain limited to material properties and cannot represent the dynamic failure processes essential for diagnosis and prognosis.

To bridge this gap, a knowledge graph schema (a representation framework that serves as a blueprint for the knowledge graph by specifying predefined types of nodes and edges) with process awareness and temporal dependency representation capabilities was designed. Meanwhile, inspired by the Case-Based Reasoning (CBR) principle, the graph is organized in a case graph-driven manner, where each graph corresponds to the complete process representation of an actual failure case. Therefore, this framework uses `BatteryCase` as the fundamental unit and establishes the following core entity and relation types:

Core entity types:

- `BatteryCase`: a complete battery sample or experiment subject
- `ProcessStep`: an experimental phase (e.g., cycling, formation)
- `Observation`: a summary point aggregating signals, failures, or metrics
- `CyclePoint`: a specific experiment time anchor (e.g., Cycle 100)

Core relation types:

- `undergoesProcess`: `BatteryCase` → `ProcessStep`
- `hasObservation`: `ProcessStep` → `Observation`
- `occursAt`: `Observation` → `CyclePoint`
- `followedBy`: `ProcessStep` → `ProcessStep`

The framework also integrates multi-dimensional entities such as key material structural attributes and multi-physics field signals (electrical, thermal, gas, mechanical). All nodes are connected via these structural relationships to form process chains, providing robust causal structure representation capabilities that support task-oriented graph retrieval and structured reasoning.

Collectively, entity nodes cover over thirty types across six major categories (Table 1), and relation types encompass structural evolution, signal attribution, and process dependency (Table 2), forming an evolution graph network that is cross-scale, multi-source information fused, and explicitly represents temporal processes. Organized case by case, this graph facilitates structural alignment, similarity retrieval, and reasoning task design, forming a foundational knowledge base that supports knowledge enhancement, question answering, mechanism attribution, and pattern discovery.

Table 1: Entity Types

**Major Category**	**Entity Type**
Core Structural Nodes	BatteryCase, ProcessStep, Observation, CyclePoint
Material and Structural Attributes	Anode Material, Cathode Material, Separator Material, Electrolyte, Additive, Cell Level Information, Porosity, Adhesion, Active Material Distribution, Areal Mass Loading, Thickness
Electrical Signals	Capacity, Specific Capacity, Energy Density, Power, Voltage, Current, SOC, SOH
Thermal / Gas / Mechanical Signals	Temperature, Internal Pressure, Gas Evolution, Mechanical Stress
Failure Analysis	Failure Form, Failure Mechanism, Failure Cause, Failure Detection Method
Monitoring and Diagnosis	Real-time Monitoring Data, Historical Maintenance, Usage Scenario

Table 2: Relation Types

**Major Category**	**Relation Type**
Core Structural Relations	undergoesProcess, hasObservation, occursAt, followedBy
Other Relations	hasMaterialComponent, hasStructureAttribute, hasCondition, inScenario, hasMonitoringData, hasSignal, hasPerformanceMetric, hasFailureForm, hasFailureMechanism, hasFailureCause, detectedBy

1.3 Text Extraction with LLMs

Traditional knowledge graph construction typically employs supervised learning models (e.g., BERT-based systems) trained on annotated corpora to extract predefined entities and relations. Such methods are inherently constrained by the fixed schema during training, as they can only recognize predetermined types and patterns, lacking adaptability to diverse expression forms found in real-world battery scientific literature.

To address this limitation, an open information extraction approach based on the DeepSeek-V3 large language model was developed, enabling efficient transformation of academic literature into structured knowledge. This method employs a hierarchical extraction strategy with a "coarse filtering → precise extraction" two-stage pipeline to ensure both efficiency and quality:

1. **Coarse Filtering Stage**: The system performs a rapid entity coverage assessment on input texts. Only documents containing relevant entity types proceed to subsequent processing, while irrelevant papers are filtered out, thereby optimizing computational resource allocation.

2. **Precise Extraction Stage**: The system operates through a carefully designed prompt engineering framework with two sequential steps:

   - *Step 1*: Guides the LLM to identify four types of core structural nodes (`BatteryCase` → `ProcessStep` → `Observation` → `CyclePoint`) and their temporal evolutionary relationships, constructing the skeletal framework of the failure process.
   - *Step 2*: Further extracts domain-specific entities, such as material components, multi-physics signals, performance metrics, and failure mechanisms, and anchors them to corresponding structural nodes through defined relationship types. This open extraction approach supports the discovery of additional entity types beyond those predefined, forming a comprehensive and extensible knowledge network.

Finally, the extraction results are output in a standardized triple-table format, with each triple containing a DOI identifier to facilitate subsequent knowledge fusion and traceability verification. Through structured prompt design and in-context learning mechanisms, the LLM captures semantic patterns beyond literal string matching, enabling it to adapt to the diverse phrasing in scientific literature. This open extraction paradigm fundamentally differs from traditional approaches by relying on the model's semantic understanding rather than predefined syntactic rules, thereby providing reliable technical support for constructing large-scale, high-quality battery failure knowledge graphs.

1.4 Knowledge Graph Data Statistics

Statistical Results: After cleaning the extraction results, we obtained the final Battery Failure Knowledge Graph (BF-KG). The statistical results show that from the initial 10,170 papers, 7,309 articles containing battery failure information were retained after filtering and underwent failure knowledge extraction. In the constructed BF-KG, a failure case is defined as a subgraph centered on a BatteryCase entity, encompassing all its associated nodes and relationships, with each case traced to its source paper via DOI. When retrieved for LLM knowledge enhancement, however, all failure cases originating from the same paper are collectively analyzed as one cohesive case study based on the paper. This approach ensures that the broader context and interrelationships between different battery cases discussed in the paper are preserved.

The structured representation of battery failure cases in BF-KG is stored in CSV files using a triplet-based format, organized into six columns: (DOI, Head Type, Head Entity, Relation Type, Tail Type, Tail Entity). An example of the knowledge graph case stored in BF-KG is shown below, while the hierarchical case structure is shown in Figure 1:

```
DOI,Head Type,Head Entity,Relation Type,Tail Type,Tail Entity
552_10...,BatteryCase,NMC Li-ion cell,hasMaterialComponent,Anode Material,Graphite
552_10...,BatteryCase,NMC Li-ion cell,hasMaterialComponent,Cathode Material,NMC811
552_10...,BatteryCase,NMC Li-ion cell,hasMaterialComponent,Electrolyte,LiFSI
552_10...,BatteryCase,NMC Li-ion cell,hasStructureAttribute,Thickness,Thick
    electrode
552_10...,BatteryCase,NMC Li-ion cell,undergoesProcess,ProcessStep,Fast Charging
552_10...,ProcessStep,Fast Charging,followedBy,ProcessStep,Cycling
552_10...,ProcessStep,Cycling,hasObservation,Observation,Capacity Fade
552_10...,Observation,Capacity Fade,hasSignal,Electrical Signal,Capacity 56.4% loss
```

```
552_10...,Observation,Capacity Fade,hasSignal,Thermal Signal,Temperature 60°C
552_10...,Observation,Capacity Fade,hasFailureForm,Failure Form,Capacity Fade
552_10...,Failure Form,Capacity Fade,hasFailureMechanism,Failure Mechanism,Lithium Plating
552_10...,Failure Mechanism,Lithium Plating,hasFailureCause,Failure Cause,Thick electrodes
552_10...,Observation,Capacity Fade,occursAt,CyclePoint,52
552_10...,BatteryCase,NMC Li-ion cell,hasMonitoringData,Monitoring Data,Thermocouple readings
552_10...,BatteryCase,NMC Li-ion cell,hasCondition,Condition,2-C charge rate
552_10...,BatteryCase,NMC Li-ion cell,hasCondition,Condition,45°C ambient temperature
552_10...,Observation,Capacity Fade,hasPerformanceMetric,Performance Metric,Cycle life 145 cycles
552_10...,BatteryCase,PCC-managed cell,hasMaterialComponent,TMS,Phase Change Composite
552_10...,BatteryCase,PCC-managed cell,hasObservation,Observation,Thermal Management
552_10...,Observation,Thermal Management,hasSignal,Thermal Signal,Temperature 50°C
552_10...,Observation,Thermal Management,hasPerformanceMetric,Performance Metric,1°C gradient
552_10...,BatteryCase,Li-metal cell,hasMaterialComponent,Anode Material,Lithium metal
552_10...,BatteryCase,Li-metal cell,hasFailureForm,Failure Form,Dendrite growth
552_10...,Failure Form,Dendrite growth,hasFailureMechanism,Failure Mechanism,Short circuit
552_10...,BatteryCase,Silicon anode cell,hasMaterialComponent,Anode Material,Silicon
552_10...,BatteryCase,Silicon anode cell,hasFailureForm,Failure Form,Volume expansion
```

```
BatteryCase: NMC-Graphite Cell
├── hasMaterialComponent
|   ├── Anode Material: Graphite
|   ├── Cathode Material: NMC622
|   └── Electrolyte: LiPF6 in EC/EMC
├── hasStructureAttribute
|   └── Areal Mass Loading: 3.2 mAh/cm²
├── hasMonitoringData
|   └── Monitoring Data: Voltage Hysteresis
├── inScenario
|   └── Usage Scenario: EV Fast Charging
└── undergoesProcess
    └── ProcessStep: Formation
        └── followedBy
            └── ProcessStep: Cycling
                ├── hasCondition
                |   ├── Condition: 1C Rate
                |   └── Condition: 45°C Ambient
                └── hasObservation
                    └── Observation: Rapid Capacity Fade
                        ├── hasSignal
                        |   ├── Electrical Signal: Capacity 80%
                        |   └── Thermal Signal: Temperature 45°C
                        ├── hasFailureForm
                        |   ├── Failure Form: Capacity Fade
                        |   |   └── hasFailureMechanism
                        |   |       └── Failure Mechanism: SEI Growth
                        |   |           └── hasFailureCause
                        |   |               └── Failure Cause: High Temperature
                        |   └── Failure Form: Swelling
                        |       └── hasFailureMechanism
                        |           └── Failure Mechanism: Gas Evolution
                        |               └── hasFailureCause
                        |                   └── Failure Cause: Electrolyte Decomposition
                        └── occursAt
                            └── CyclePoint: 300
```

Figure 1: **Example of the hierarchical case structure in BF-KG**.

The core statistical results of the constructed BF-KG are summarized below, with the top 30 entity types and their counts detailed in Table 3:

- Total entity nodes: 181,694
- Total relationship edges: 177,520
- Total failure cases: 11,814
- Average nodes per paper: 24.86
- Maximum entities per paper: 70
- Average edges per paper: 24.29
- Maximum edges per paper: 67
- Average length of the longest path (number of nodes): 13.55
- Maximum length of the longest path (number of nodes): 32

Table 3: Entity Types and Representative Entities in BF-KG (Top 30)

Entity Type	Count	Representative Entities
Observation	18,552	Capacity Fade, Capacity Retention, Initial Performance, SEI Formation, Thermal Runaway
ProcessStep	17,119	Cycling, Formation, Charging, Discharging, Lithiation
CyclePoint	12,398	100, 1, 50, 500, 200
Electrical Signal	12,157	Voltage Drop, Capacity 80%, Voltage 4.2V, Voltage 3.7V, Current 2A
BatteryCase	11,814	Li-ion Battery, Li-S Battery, LiFePO4, NMC-Graphite, Pouch Cell
Failure Mechanism	10,868	SEI Growth, Lithium Plating, Volume Change, Electrolyte Decomposition, Mechanical Stress
Failure Form	10,509	Capacity Fade, Thermal Runaway, Structural Degradation, Short Circuit, Cracking
Failure Cause	8,840	High Temperature, Volume Change, Side Reactions, Overcharging, Lithiation/Delithiation
Monitoring Data	8,555	Voltage Profile, SEM Imaging, EIS, Temperature, XRD Analysis
Cathode Material	7,415	$LiCoO_2$, $LiFePO_4$, NMC, $LiMn_2O_4$, Sulfur
Anode Material	7,206	Graphite, Lithium Metal, Silicon, Carbon, Lithium Foil
Electrolyte	6,244	$LiPF_6$ in EC/DMC, $LiPF_6$, $LiPF_6$ in EC/DEC, Organic Carbonate, Sulfuric Acid
Performance Metric	4,915	SOH, RUL, Capacity Fade, Cycle Life, Energy Density
Thermal Signal	4,767	Temperature 60°C, Temperature Rise, Temperature 45°C, Room Temperature, Temperature 25°C
Failure Detection Method	4,513	SEM, EIS, XRD, XPS, TEM
Usage Scenario	4,018	Electric Vehicles, Fast Charging, Portable Electronics, Grid Storage, EV Battery Pack
Capacity	3,144	80% of initial, Reduced, 140 mAh/g, 150 mAh/g, 120 mAh/g
Mechanical Signal	2,889	Volume Expansion, Tensile Stress, Stress, Compressive Stress, Radial Stress
Thickness	2,885	Standard, Variable, 50 µm, Nanoscale, 100 µm
Porosity	2,580	High, 30%, Low, Mesoporous, Controlled
Condition	2,401	Temperature 25°C, Room Temperature, High Temperature, 1C Rate, High C-rate
Separator Material	2,183	Celgard 2400, Polyethylene, Polypropylene, Polyolefin, Celgard 2325
Additive	1,765	FEC, $LiNO_3$, Vinylene Carbonate, $LiPF_6$, Carbon Black
Structure Attribute	1,397	Internal Resistance, Cylindrical Cell, Porosity, 400%, Pouch Cell
Capacity Retention	1,318	80%, 90%, 95%, 86%, 75%
Specific Capacity	1,174	150 mAh/g, 4200 mAh/g, 170 mAh/g, 372 mAh/g, 200 mAh/g
Areal Mass Loading	1,122	3.5 mAh/cm^2, 3.2 mAh/cm^2, 3 mg/cm^2, 1 mg/cm^2, 2 mg/cm^2
Efficiency	1,009	99%, 98%, ˜100%, 99.5%, 95%
Cell Level Information	849	Cylindrical, Prismatic, Pouch, 18650, Series-connected
Binder	744	PVDF, CMC, PAA, PTFE, Alginate

Failure Chain Statistics: To gain insights into the internal structure of the constructed BF-KG and understand the causal failure relationships embedded within it, a systematic statistics of failure chains in the graph was conducted. This statistics aims to reveal the typical causal propagation patterns of ”Failure Form → Failure Mechanism → Failure Cause”.

The statistical process began by identifying the top 30 most frequent entities for three failure related categories: Failure Form, Failure Mechanism, and Failure Cause. The selection was based on occurrence frequency across all cases in BF-KG, ensuring the analysis focuses on statistically significant core entities. Subsequently, complete failure chains were identified within BF-KG cases, requiring that the same failure mechanism entity connects to both a failure form and a failure cause, forming meaningful three-node causal paths. Throughout this process, only the top 30 high-frequency entities from each category were considered to maintain chain representativeness. Finally, the top 20 most frequent complete failure chains were selected based on their case occurrence frequency and decomposed into edge relationships for frequency statistics. The output provides edge list data suitable for network visualization and in-depth analysis.

Beyond the global statistics, a comparative analysis was conducted across different cathode material systems to investigate material-specific failure characteristics. Based on the cathode material involved in each case, the dataset was divided into three major categories: NMC (Nickel-Manganese-Cobalt oxide), LFP (Lithium Iron Phosphate), and LCO (Lithium Cobalt Oxide). The same statistical methodology was applied to each subset to identify the dominant failure chains for each material system. The statistical results of these failure chains are presented in Table 4 (global Top 20) and Tables 5-7 (material-specific Top 10).

Table 4: Global Failure Chains in BF-KG (Top 20)

**Failure Chain**	**Count**
Capacity Fade → SEI Growth → High Temperature	193
Capacity Fade → Lithium Plating → Overcharging	146
Capacity Fade → Volume Change → Lithiation/Delithiation	63
Capacity Fade → SEI Growth → Electrolyte Decomposition	60
Capacity Fade → Lithium Plating → Low Temperature	33
Cracking → Mechanical Stress → Volume Change	27
Capacity Fade → Particle Isolation → Volume Change	27
Short Circuit → Dendrite Growth → Lithium Plating	26
Capacity Fade → Structural Degradation → Volume Change	26
Capacity Fade → SEI Growth → Side Reactions	25
Capacity Fade → Pulverization → Volume Change	25
Capacity Fade → SEI Growth → Repeated Cycling	24
Cracking → Volume Change → Lithiation/Delithiation	24
Capacity Fade → SEI Formation → Electrolyte Decomposition	21
Capacity Fade → Polysulfide Shuttle → Side Reactions	21
Capacity Fade → Lithium Plating → High Charge Rate	20
Capacity Fade → SEI Decomposition → Volume Change	20
Capacity Fade → Aging → High Temperature	19
Capacity Fade → Aging → Repeated Cycling	18
Capacity Fade → Active Material Loss → Side Reactions	17

Analysis of Material-Specific Failure Characteristics: The comparative analysis reveals distinct failure patterns across different cathode material systems:

NMC System (High-Temperature Sensitivity, SEI-Dominated): The NMC system exhibits a strong dominance of SEI-related failures, particularly under high-temperature conditions. The chain “Capacity Fade → SEI Growth → High Temperature” appears 154 times, accounting for 20.6% of all complete chains in this system. SEI-related chains appear four times in the Top 10 with a total frequency of 174, demonstrating that accelerated SEI growth at elevated temperatures is the primary degradation mechanism for NMC-based batteries. This aligns with the known thermal sensitivity of NMC materials.

LFP System (Overcharge Sensitivity, Thermal Stability): The LFP system shows distinct overcharge-related failure patterns. The chain “Capacity Fade → Lithium Plating → Overcharging” ranks first with 34 occurrences, and overcharge/lithium plating related chains account for 46% of the Top 10 frequencies. Notably, high-temperature SEI growth (18 occurrences) is significantly less frequent compared to NMC (154

Table 5: Top 10 Failure Chains for NMC System

**Failure Chain**	**Count**
Capacity Fade → SEI Growth → High Temperature	154
Capacity Fade → Lithium Plating → Overcharging	37
Capacity Fade → Lithium Plating → Low Temperature	20
Swelling → Gas Evolution → Electrolyte Decomposition	13
Capacity Fade → Lithium Plating → High Charge Rate	11
Capacity Fade → SEI Growth → Side Reactions	9
Capacity Fade → Lithium Plating → High Current Density	7
Thermal Runaway → Internal Short Circuit → High Temperature	6
SEI Growth → Electrolyte Decomposition → High Temperature	6
Capacity Fade → SEI Growth → High SOC	5

Table 6: Top 10 Failure Chains for LFP System

**Failure Chain**	**Count**
Capacity Fade → Lithium Plating → Overcharging	34
Capacity Fade → SEI Growth → High Temperature	18
Capacity Fade → SEI Growth → Electrolyte Decomposition	14
Capacity Fade → Active Material Loss → SEI Growth	10
Capacity Fade → Active Material Loss → Side Reactions	8
Capacity Fade → Lithium Plating → Low Temperature	8
Capacity Fade → SEI Growth → Repeated Cycling	6
Thermal Runaway → Electrolyte Decomposition → High Temperature	6
Cracking → Mechanical Stress → Volume Change	5
Capacity Fade → Active Material Loss → Lithium Plating	5

Table 7: Top 10 Failure Chains for LCO System

**Failure Chain**	**Count**
Capacity Fade → Lithium Plating → Overcharging	52
Capacity Fade → SEI Growth → Electrolyte Decomposition	29
Capacity Fade → SEI Growth → High Temperature	22
Capacity Fade → Structural Degradation → Volume Change	12
Capacity Fade → Lithium Plating → Low Temperature	9
Short Circuit → Dendrite Growth → Lithium Plating	9
Capacity Fade → SEI Growth → Repeated Cycling	8
Capacity Fade → SEI Growth → Side Reactions	7
Capacity Fade → Active Material Loss → Lithium Plating	6
Thermal Runaway → Dendrite Growth → Overcharging	6

occurrences), confirming the superior thermal stability of LFP chemistry. The results suggest that overcharge safety, rather than thermal degradation, is the primary concern for LFP-based batteries.

LCO System (Structural Instability, Diverse Failure Modes): The LCO system exhibits the highest failure mode diversity with 419 unique chains. Distinctive features include structural degradation-related failures (e.g., “Capacity Fade → Structural Degradation → Volume Change” with 12 occurrences) and dendrite/short circuit issues (e.g., “Short Circuit → Dendrite Growth → Lithium Plating” with 9 occurrences). These patterns reflect the structural instability of LCO under high-voltage operation and its tendency toward lithium dendrite formation. The even distribution of failure frequencies (no single chain exceeding 7% of total) further confirms the diverse degradation behavior of LCO materials.

Section 2. KAG-based Battery Failure Diagnosis Method

2.1 Failure Feature-Aware Retrieval Algorithm

The application of LLMs to battery failure diagnosis is constrained by their limited domain knowledge. To bridge this gap, the Battery Failure Knowledge Augmented Generation (BF-KAG) framework is introduced. Its core relies on retrieving pertinent failure cases to inform the diagnostic process, for which the Failure Feature-Aware Case Graph Retrieval Algorithm is proposed. The retrieval algorithm operates through the following key stages to identify the most relevant failure cases from BF-KG based on input experimental data:

Input: Natural language description D of battery failure scenario, case base $C = \{c_1, c_2, \ldots, c_N\}$, top-$K$ parameter, diagnostic targets $\mathscr{T}$ (e.g., `Failure Mechanism`, `Failure Cause`), and embedding model M.

Query Case Construction The process begins by converting natural language descriptions of battery materials, experimental conditions, and observed failure phenomena into a structured case graph, employing the same method used in BF-KG construction. This conversion ensures optimal alignment with the BF-KG structure for subsequent retrieval.

Specifically, the description D is first parsed into target triples, which serve as the fundamental building blocks for constructing the case graph:

$$T = \{t_1, t_2, \ldots, t_N\} = \text{CaseGraphBuild}(D)$$

These triples are then preprocessed into textual representations to enable effective vectorization for efficient retrieval. Using the FormatTriplet2Text function, each structured triple is converted into a natural language sentence following the template: "The `[head_type]` '`[head_entity]`' `--[relation_type]-->` `[tail_type]` '`[tail_entity]`'."

$$\text{texts} = [\text{FormatTriplet2Text}(t_i) \quad \forall t_i \in T]$$

To enable fine-grained retrieval at the triplet level, each textual representation is individually encoded into semantic embeddings using the embedding model M, which is implemented with SentenceTransformer:

$$E_t = M(\text{texts}) \in \mathbb{R}^{N \times d}$$

where d is the embedding dimension.

Target-Specific Case Filtering This step pre-filters the case base to retain only cases containing the desired types of diagnostic conclusions, ensuring that retrieved cases possess direct comparability and diagnostic relevance for the target analysis. For each candidate case $c_j \in C$, the algorithm checks if it contains any triple with tail type in the diagnostic targets $\mathscr{T}$:

$$\text{filter}(c_j) = \begin{cases} \text{true} & \text{if } \exists t \in c_j.\text{triples} : t.\text{tail\_type} \in \mathscr{T} \\ \text{false} & \text{otherwise} \end{cases}$$

Only cases satisfying this condition proceed to subsequent stages.

Fine-Grained Semantic Matching This stage performs detailed similarity computation at the triplet level, capturing semantic correspondences between the query case and candidate cases through embedding-based cosine similarity. For each filtered case c_j, its precomputed embeddings $E_c^j \in \mathbb{R}^{M_j \times d}$ are retrieved, where M_j denotes the number of triples in case c_j and d is the embedding dimension. The similarity matrix $\text{matrix}_j \in \mathbb{R}^{N \times M_j}$ between query and case triplets is computed as:

$$\text{matrix}_j = \text{CosineSimilarity}(E_t, E_c^j)$$

where N is the number of query triples, and each element $\text{matrix}_j[i,k] = \frac{E_t[i] \cdot E_c^j[k]}{\|E_t[i]\| \|E_c^j[k]\|}$ represents the cosine similarity between the i-th query triple and k-th case triple.

Diagnostic Relevance Aggregation This phase aggregates triplet-level similarity scores into case-level relevance scores, employing a max-pooling strategy to identify the best matching evidence for each query aspect. For each query triplet t_i, the maximum similarity score with any triplet in case c_j is identified, denoted

as $s_i^{\max}$:

$$s_i^{\max} = \max_{k=1}^{M_j} \text{matrix}_j[i,k]$$

The overall case similarity score S_j for case c_j is obtained by summation:

$$S_j = \sum_{i=1}^{N} s_i^{\max}$$

Case Ranking and Retrieval Finally, candidate cases are ranked by their aggregated similarity scores. Let J denote the set of indices of filtered cases. The sorted indices are obtained by:

$$j_1, j_2, \ldots, j_{|J|} = \text{argsort}\left(\{S_j\}_{j\in J}, \text{descending}\right)$$

The top-K cases form the retrieved case set $C_{\text{retrieved}}$:

$$C_{\text{retrieved}} = \{c_{j_1}, c_{j_2}, \ldots, c_{j_K}\}$$

This set $C_{\text{retrieved}}$ constitutes the final output of the retrieval algorithm, providing the most relevant and diagnostically valuable failure cases as critical references for the LLM.

The complete retrieval workflow integrates semantic matching with target-specific filtering to ensure both relevance and diagnostic value. The algorithm efficiently identifies the most pertinent failure cases by combining fine-grained triplet-level similarity computation with case-level relevance aggregation. The overall procedure is summarized in Algorithm 1.

Algorithm 1 Failure Feature-Aware Case Graph Retrieval Algorithm

Input: Natural language description D, case base C, top-K, diagnostic targets $\mathscr{T}$, embedding model M
Output: Retrieved case set $C_{\text{retrieved}}$
Parse description to triples: $T \leftarrow \text{CaseGraphBuild}(D)$
Preprocess triples to text: texts $\leftarrow [\text{FormatTriplet2Text}(t_i) \quad \forall t_i \in T]$
Encode text to embeddings: $E_t \leftarrow M(\text{texts})$
Initialize score list $S \leftarrow [\,]$
for each case $c_j \in C$ **do**
  if $\nexists t \in c_j$.triples such that t.tail_type $\in \mathscr{T}$ **then**
    continue to next case
  end if
  Retrieve case embeddings $E_c^j \leftarrow c_j$.triple_embeddings
  Compute similarity matrix: $\text{matrix}_j \leftarrow \text{CosineSimilarity}(E_t, E_c^j)$
  Compute max scores: $s_i^{\max} \leftarrow \max_k \text{matrix}_j[i,k] \quad \forall i$
  Compute total score: $S_j \leftarrow \sum_i s_i^{\max}$
  S.append$((S_j, c_j))$
end for
Sort S by S_j descending
$C_{\text{retrieved}} \leftarrow \{c_j \mid (S_j, c_j) \in S[1:K]\}$
Return $C_{\text{retrieved}}$

2.2 Task Prompt Build for Battery Failure Diagnosis

Following the retrieval of relevant failure cases, we construct a structured prompt to guide the LLM in performing battery failure diagnosis. The prompt engineering process integrates the retrieved cases with the original query to form a comprehensive context for knowledge-augmented generation.

Prompt Structure The prompt is organized into several key components (using the failure cause and mechanism diagnosis task as an example):

- **System Prompt**: Defines the AI assistant's role as a battery failure analysis specialist and specifies the task constraints, including the requirement to select answers only from provided candidate labels. The system prompt is constructed as follows:

```
You are a scientific assistant specialized in battery materials and failure analysis.
Given structured experimental observations and material properties, your task is to
infer the top possible failure causes and failure mechanisms of the battery system.
```

- **Analysis Case**: Presents the structured experimental observations and material properties of the target battery case, formatted using the same FormatTriplet2Text function employed during retrieval. This section provides the core context for diagnosis:

```
The BatteryCase "NMC Battery" --hasMaterialComponent--> Cathode Material "NMC".
The BatteryCase "NMC Battery" --undergoesProcess--> ProcessStep "Cycling Test".
The ProcessStep "Cycling Test" --hasObservation--> Observation "Capacity Fade".
The Observation "Capacity Fade" --occursAt--> CyclePoint "589".
The Observation "Capacity Fade" --hasPerformanceMetric--> Performance Metric "90
...
```

- **Reference Cases**: When retrieval is enabled, this section provides up to *K* relevant historical failure cases from BF-KG, each formatted with its DOI identifier and structured triples converted to natural language text using the FormatTriplet2Text function, ensuring consistent representation with the analysis case. Example reference cases include:

```
Related Historical Cases
Case1:
The BatteryCase "NMC" --hasMaterialComponent--> Cathode Material "Nickel Manganese
Cobalt Oxide".
The BatteryCase "NCA" --hasMaterialComponent--> Cathode Material "Nickel Cobalt
Aluminum Oxide".
...
The Failure Form "Capacity Fade" --hasFailureMechanism--> Failure Mechanism "Lithium
Plating".
The Failure Mechanism "Lithium Plating" --hasFailureCause--> Failure Cause "Low
Temperature".
Case2:
The BatteryCase "C/NMC" --hasMaterialComponent--> Anode Material "Graphite-Silicon
Alloy".
...
The Failure Form "Capacity Fade" --hasFailureMechanism--> Failure Mechanism "Lithium
Plating".
The Failure Mechanism "Lithium Plating" --hasFailureCause--> Failure Cause "Low
Temperature Cycling".
...
```

- **Candidate Labels**: Explicitly lists the available failure causes and failure mechanisms for the model to choose from, ensuring constrained generation. The candidate lists are presented as:

```
Candidate Failure Causes:
Aging, Electrolyte Decomposition, High Temperature, Low Temperature, Overcharging, ...
Candidate Failure Mechanisms:
Lithium Plating, SEI Growth, Structural Degradation, Transition Metal Dissolution, ...
```

- **Task Instruction**: Clearly specifies the diagnostic objective to identify the top-K most likely failure causes and mechanisms, with explicit formatting requirements. The task instruction directs the LLM as follows:

```
Based on the battery structure and observation data provided above, as well as the
related historical cases, please infer the top-1 most likely Failure Causes and Failure
Mechanisms of the system.
Answer in the following JSON format:
"Failure Cause":  ["..."],
"Failure Mechanism":  ["..."]
```

Adaptive Prompting The prompt structure dynamically adapts based on the availability of retrieved cases:

$$\text{Prompt} = \begin{cases} \text{Analysis Case} + \text{Reference Cases} + \text{Candidate Labels} + \text{Task Instruction} & \text{if } C_{\text{retrieved}} \neq \emptyset \\ \text{Analysis Case} + \text{Candidate Labels} + \text{Task Instruction} & \text{otherwise} \end{cases}$$

Where $C_{\text{retrieved}} \neq \emptyset$ indicates that relevant cases were successfully retrieved from BF-KG, enabling the enhanced prompt structure with reference cases. When no relevant cases are found ($C_{\text{retrieved}} = \emptyset$), the prompt relies solely on the analysis case and candidate labels. The task instruction adapts accordingly, with the enhanced version specifically referencing the historical cases as additional evidence for the diagnostic reasoning process. This structured prompting approach ensures that the LLM leverages both the immediate experimental data and relevant historical knowledge from BF-KG to generate mechanistically grounded failure diagnoses while maintaining output consistency through constrained generation.

Notably, the task instruction can be flexibly extended beyond basic output formats to incorporate additional elements such as explanatory reasoning, supporting evidence, and mitigation recommendations through modular component selection. While the current implementation utilizes a simplified JSON structure for clarity, the underlying framework readily accommodates expanded output formats that include diagnostic rationales and preventive suggestions when required by specific application scenarios.

2.3 Illustrative Example of the Knowledge-Augmented LLM Diagnosis Process

To provide a concrete understanding of how BattFailScholar processes a battery failure query through the failure feature-aware retrieval algorithm to construct the final augmented prompt, we present a step-by-step walkthrough using a representative example.

Step 1: Input Query Description

Consider a researcher investigating a capacity fade issue in an NMC811/graphite lithium-ion battery after extensive cycling. The raw input description is:

> "NMC811 battery subjected to extensive cycling at room temperature exhibits significant capacity fade after 589 cycles, with current state of health at 90%. Post-mortem analysis reveals lithium plating on the anode surface."

Step 2: Query Case Graph Construction

This natural language description is parsed into structured triples following the BF-KG schema, resulting in the query case graph shown in Table 8. Each triple is then converted to a textual representation.

Step 3: Target-Specific Filtering

The diagnostic targets are set to $\mathscr{T} = \{\text{Failure Mechanism}, \text{Failure Cause}\}$. The algorithm scans the BF-KG case base and retains only those cases containing triples with tail types matching these targets. Cases lacking any failure-related conclusions are filtered out.

Step 4: Triplet-Level Semantic Matching

Each textual triple from the query is encoded into embeddings using the sentence transformer model. For a candidate case c_j containing M_j triples, a similarity matrix $\text{matrix}_j \in \mathbb{R}^{7 \times M_j}$ is computed, where each entry represents the cosine similarity between a query triple and a case triple.

Table 8: Structured Triples Extracted from Input Query

Head Type	Head Entity	Relation	Tail Type/Tail Entity
BatteryCase	NMC Battery	hasMaterialComponent	Cathode Material / NMC
BatteryCase	NMC Battery	hasMaterialComponent	Anode Material / Graphite
BatteryCase	NMC Battery	undergoesProcess	ProcessStep / Cycling Test
ProcessStep	Cycling Test	hasObservation	Observation / Capacity Fade
Observation	Capacity Fade	occursAt	CyclePoint / 589
Observation	Capacity Fade	hasPerformanceMetric	Performance Metric / 90% SOH
Observation	Capacity Fade	hasPostMortemFinding	Finding / Lithium Plating

For illustration, consider a retrieved case with DOI "10.1149/2.0121908jes" describing an NMC/graphite cell that experienced lithium plating under similar conditions. The top matching triple pairs might include:

- Query triple: "Observation 'Capacity Fade' –hasPostMortemFinding–→ Finding 'Lithium Plating" matches Case triple: "Observation 'Capacity Loss' –hasPostMortemFinding–→ Finding 'Lithium Deposition" (similarity: 0.92)
- Query triple: "BatteryCase 'NMC Battery' –hasMaterialComponent–→ Cathode Material 'NMC" matches Case triple: "BatteryCase 'NMC622 Cell' –hasMaterialComponent–→ Cathode Material 'NMC"' (similarity: 0.89)

Step 5: Relevance Aggregation and Ranking with Failure Feature-Aware Design

For each query triple t_i, the maximum similarity $s_i^{\max}$ across all case triples is identified. The overall case similarity score $S_j = \sum_{i=1}^{7} s_i^{\max}$ is then computed.

A key distinction of our retrieval approach lies in this aggregation strategy. Rather than averaging similarities across all triples, which could be dominated by non-diagnostic information such as material specifications or experimental conditions, our max-pooling approach identifies the best matching evidence for each query aspect. This ensures that a case is considered relevant if it contains at least some highly relevant diagnostic information, even if other aspects differ. Combined with the target-specific filtering in Step 3, which retains only cases containing diagnostic conclusions relevant to the task, this failure feature-aware design addresses a fundamental challenge in battery failure retrieval: cases that appear superficially similar based on materials or operating conditions may involve entirely different failure mechanisms, while diagnostically valuable cases with different base materials might share the same underlying degradation pathway.

After processing all candidate cases, the top-K cases with highest S_j are selected. For this example, suppose the top-2 retrieved cases are:

- Case A (DOI: 10.1149/2.0121908jes): NMC622/graphite cell with lithium plating after low-temperature cycling (similarity score: 6.42)
- Case B (DOI: 10.1002/batt.202300123): NMC811/graphite cell with capacity fade attributed to SEI growth (similarity score: 5.87)

Step 6: Constructing the Augmented Prompt

The retrieved cases are integrated with the original query to form the final prompt. Figure 2 illustrates the complete prompt structure for this example.

Step 7: LLM Response and Diagnosis

The LLM processes this augmented prompt and generates a response. For this example, a plausible output would be:

System Prompt:
You are a scientific assistant specialized in battery materials and failure analysis. Given structured experimental observations and material properties, your task is to infer the top possible failure causes and failure mechanisms of the battery system.
Analysis Case:
The BatteryCase 'NMC Battery' → hasMaterialComponent → Cathode Material 'NMC'.
The BatteryCase 'NMC Battery' → hasMaterialComponent → Anode Material 'Graphite'.
The BatteryCase 'NMC Battery' → undergoesProcess → ProcessStep 'Cycling Test'.
The ProcessStep 'Cycling Test' → hasObservation → Observation 'Capacity Fade'.
The Observation 'Capacity Fade' → occursAt → CyclePoint '589'.
The Observation 'Capacity Fade' → hasPerformanceMetric → Performance Metric '90% SOH'.
The Observation 'Capacity Fade' → hasPostMortemFinding → Finding 'Lithium Plating'.
Related Historical Cases:
Case 1 (DOI: 10.1149/2.0121908jes):
The BatteryCase 'NMC622 Cell' → hasMaterialComponent → Cathode Material 'NMC'.
...
The Observation 'Capacity Loss' → hasPostMortemFinding → Finding 'Lithium Deposition'.
The Failure Form 'Capacity Loss' → hasFailureMechanism → Failure Mechanism 'Lithium Plating'.
The Failure Mechanism 'Lithium Plating' → hasFailureCause → Failure Cause 'Low Temperature Cycling'.
Case 2 (DOI: 10.1002/batt.202300123):
The BatteryCase 'NMC811 Pouch Cell' → hasMaterialComponent → Cathode Material 'NMC'.
...
The Observation 'Capacity Fade' → hasPostMortemFinding → Finding 'SEI thickening'.
The Failure Form 'Capacity Fade' → hasFailureMechanism → Failure Mechanism 'SEI Growth'.
The Failure Mechanism 'SEI Growth' → hasFailureCause → Failure Cause 'Aging'.
Candidate Labels:
Failure Causes: Aging, Electrolyte Decomposition, High Temperature, Low Temperature, Overcharging, Overdischarge, SEI Formation, Short Circuit, Side Reactions, Volume Change
Failure Mechanisms: Dendrite Growth, Electrolyte Decomposition, Gas Evolution, Internal Short Circuit, Lithium Plating, Phase Transition, SEI Decomposition, SEI Growth, Structural Degradation, Thermal Runaway
Task Instruction:
Based on the battery structure and observation data provided above, as well as the related historical cases, please infer the top-1 most likely Failure Causes and Failure Mechanisms of the system.
Answer in the following JSON format: { "Failure Cause": ["..."], "Failure Mechanism": ["..."] }

Figure 2: Complete augmented prompt constructed from the example query and retrieved cases.

```
{
  "Failure Cause": ["Low Temperature Cycling"],
  "Failure Mechanism": ["Lithium Plating"]
}
```

This diagnosis correctly identifies lithium plating as the failure mechanism and low-temperature cycling as the contributing cause, leveraging the evidence from Case 1 which reported similar post-mortem findings. The framework successfully retrieves and utilizes relevant historical knowledge to enhance diagnosis.

Section 3. Long-Tail Problem of Failure Types and Reliable Risk Assessment

3.1 Drift-Aware Correction Mechanism

The drift-aware correction mechanism is designed to address the long-tail problem in battery failure diagnosis, where low-frequency failure types suffer from poor diagnostic reliability due to scarce samples in the case base. The mechanism detects and corrects erroneous KAG-enhanced predictions by monitoring two key indicators: label distribution shifts and chemical system shifts.

Detection of Samples Requiring Revision

The correction process begins by analyzing prediction changes between base LLM and KAG-enhanced outputs. For each sample, the number of changed labels within the top-k predictions is calculated as:

$$\text{change\_count} = k - |\text{LLM\_pred}_{[:k]} \cap \text{KAG\_pred}_{[:k]}|$$

Samples with change_count exceeding a threshold (typically 3 out of 5) are identified as candidates for potential revision. For these candidates, further analysis is conducted to determine whether the prediction shift represents a genuine improvement or a degradation caused by noisy retrieved cases.

Frequency-Based Grouping and Analysis

Failure types are categorized into high-frequency and low-frequency groups based on their occurrence counts in the case base. Types with fewer than 100 occurrences are defined as low-frequency. Statistical analysis of prediction changes across these groups reveals distinct patterns:

- For high-frequency types, substantial prediction changes after KAG enhancement typically correspond to effective error correction, as sufficient relevant cases provide reliable reference information.
- For low-frequency types, substantial prediction changes often lead to misclassification, as retrieved references are predominantly noisy due to scarce samples.

Chemical System Shift Detection

To further differentiate between beneficial corrections and harmful noise introductions, the mechanism monitors shifts in chemical system composition.

Chemical System Definition Failure types are categorized into distinct chemical systems based on their underlying degradation pathways. For failure cause analysis, the system defines four primary categories:

- **Thermal/Electrical Abuse**: Includes overcharging, high temperature, high current density, and short circuit
- **Aging/Side Reactions**: Includes aging, side reactions, SEI formation, unstable SEI, and repeated cycling
- **Mechanical/Structural**: Includes volume change and mechanical stress
- **Extreme Potential**: Includes overdischarge, low temperature, high voltage operation, and electrolyte decomposition

For failure mechanism analysis, the system defines six primary categories:

- **Thermal Abuse**: Includes thermal runaway, overheating, and exothermic reactions
- **Electrical Abuse**: Includes lithium plating, dendrite growth, and internal short circuit
- **Mechanical Degradation**: Includes volume change, mechanical stress, structural degradation, active material loss, and phase transition

- **Electrochemical Side Reactions**: Includes SEI growth, SEI decomposition, electrolyte decomposition, side reactions, gas evolution, and transition metal dissolution
- **Aging/Degradation**: Includes aging and general degradation
- **Chemistry-Specific**: Includes polysulfide shuttle and other material-specific mechanisms

For each sample, the dominant chemical system in base predictions and KAG predictions is identified using the predefined failure-system mappings described above. A significant system shift is detected when:

- The dominant system in base predictions differs from that in KAG predictions, or
- Multiple new systems are introduced in KAG predictions that were absent in base predictions

Revision Decision Logic

A sample is flagged for revision when both conditions are met, indicating that the prediction shift is likely harmful rather than beneficial:

1. **Significant label distribution shift**: change_count $\geq$ threshold (typically 3 out of 5). This indicates that KAG enhancement substantially altered the prediction set compared to base LLM.
2. **Substantial chemical system shift**: The KAG predictions introduce new chemical systems that conflict with the base prediction's system distribution. This is detected when the dominant system in base predictions differs from that in KAG predictions, or when multiple new systems appear in KAG predictions that were absent in base predictions.

The combination of these two indicators serves as a reliable proxy for identifying cases where low-frequency types are misclassified due to noisy retrieved cases. For low-frequency types, substantial label changes alone often result from random noise, but when coupled with significant system shifts, it indicates cross-system contamination where retrieved cases from different chemical systems introduce conflicting information. In contrast, for high-frequency types, even substantial label changes typically maintain system consistency, reflecting genuine corrections supported by sufficient relevant cases.

For flagged samples, the mechanism reverts to the more conservative base LLM predictions. The revision decision follows specific patterns based on the target type:

- **Failure Cause**: Patterns include low-frequency dominated base predictions transitioning to high-frequency dominated KAG predictions with different systems, and all-low-frequency base predictions introducing multiple new high-frequency labels from new systems.
- **Failure Mechanism**: Patterns include base predictions with moderate low-frequency counts introducing multiple high-frequency labels from different new systems, and low-frequency dominated base predictions shifting to high-frequency dominated KAG predictions with new system introduction.

Implementation Options

The mechanism supports two revision modes:

- **Direct Revision Mode**: Flagged samples are directly replaced with base LLM predictions. This mode is simple and effective when the detection logic is highly reliable.
- **LLM-Assisted Revision Mode**: For flagged samples, a separate LLM query is invoked to compare base and KAG predictions, selecting the more plausible one based on experimental observations and degradation mechanisms. The prompt includes the original triples, both predictions, candidate labels, and retrieved cases, requesting a JSON-formatted decision with reasoning.

Analysis of LLM-assisted revision reveals that when presented with only the two prediction options, the model tends to favor base predictions (internal knowledge). However, when KAG-retrieved cases are also provided for comparison, the model shows increased preference for contextually grounded predictions. Given this observation and the additional computational overhead and response latency introduced by extra LLM queries, Direct Revision Mode is recommended as the primary implementation. Experimental results validate its effectiveness while avoiding the time cost of additional inference requests.

3.2 Pattern Matching-Based Voting Algorithm

Pattern Matching-Based Voting Mechanism for Risk Assessment

The pattern matching-based voting mechanism is designed to provide reliable risk level estimation by integrating diagnostic information from multiple candidate failure causes and mechanisms. This approach addresses the limitation of conventional LLM-based methods that tend to produce uniformly high confidence scores with limited discriminative power.

Failure-Risk Pattern Library Construction

The first step involves constructing a 'Failure-Risk' pattern library by statistically analyzing correlations between failure factors (causes, mechanisms, forms) and their associated risk levels. Using the ground truth data, patterns are extracted at multiple granularity levels:

- **Single-factor patterns**: Risk distributions for individual failure causes, mechanisms, or forms.
- **Two-factor patterns**: Risk distributions for combinations of cause+mechanism, cause+form, and mechanism+form.
- **Three-factor patterns**: Risk distributions for complete combinations of cause+mechanism+form.

For each pattern, the dominant risk level is identified when its occurrence frequency exceeds a confidence threshold (typically 60%) with a minimum sample count (at least 2 samples). The resulting pattern library serves as a prior knowledge base for subsequent risk inference.

Multi-Candidate Risk Inference

During risk assessment, the Top-N predicted failure causes and mechanisms (ranked by probability from the diagnostic module) are used as input. For a given sample with failure form f, failure cause candidates $C = [c_1, c_2, ..., c_N]$ (ordered by decreasing probability), and failure mechanism candidates $M = [m_1, m_2, ..., m_N]$, the mechanism systematically explores all combinations within the top K candidates (typically $K = 3$):

1. **Three-factor matching**: Attempt to match complete patterns $(c_i + m_j + f)$ from the three-factor rule library.
2. **Two-factor matching**: If no three-factor match is found, attempt matches for $(c_i + m_j)$, $(c_i + f)$, and $(m_j + f)$ from the two-factor rule library.
3. **Single-factor matching**: If no multi-factor matches are found, fall back to individual matches for c_i, m_j, or f from the single-factor rule library.

Weighted Voting Scheme

Each matched pattern contributes a weighted vote to its associated risk level. The vote weight is computed as:

$$w = \alpha \times \beta \times \gamma$$

where:

- α is the pattern type weight (3.0 for three-factor, 2.5 for two-factor, 2.0 for single-factor)
- β is the pattern confidence from the library (0-1)
- γ is a rank-based factor that accounts for candidate probabilities: $\gamma = \prod(1/\text{rank}_i)$ for all factors in the pattern

The total votes for each risk level $r \in \{L1, L2, L3\}$ are aggregated as:

$$\text{votes}[r] = \sum_{\text{patterns matching } r} w_{\text{pattern}}$$

The final risk level is determined as the one with the highest accumulated votes:

$$\text{risk}_{\text{final}} = \arg\max_{r} \text{votes}[r]$$

The normalized confidence score is calculated as:

$$\text{confidence} = \frac{\text{votes}[\text{risk}_{\text{final}}]}{\sum_r \text{votes}[r]}$$

The mechanism also records supporting evidence, including the top contributing patterns with their factors, confidence scores, and vote weights, providing interpretable justification for the risk assessment.

Integration with LLM-Based Risk Assessment

For comparison purposes, a pure LLM-based risk assessment is also implemented, where the model directly predicts risk levels from the top-K failure candidates. The prompt includes the battery structure and observation data, the ranked candidate lists, and risk level definitions, requesting a JSON response with both the predicted risk level and a confidence score (0-100).

Analysis reveals distinct characteristics of the two approaches:

- The LLM-based method tends to produce uniformly high confidence scores concentrated in a narrow range (70-80%), offering limited discriminative power.
- The pattern-based voting method yields a more reasonable confidence distribution where higher confidence scores correlate with better actual accuracy.
- For high-risk (L3) predictions, the LLM-based method demonstrates relatively good accuracy when producing high-confidence outputs.

Hybrid Approach

Based on these observations, a hybrid strategy is implemented that combines the strengths of both methods. The pattern-based voting results serve as the foundation, with selective replacement for cases where:

- The LLM predicts L3 (high-risk) with confidence $\geq 80\%$

Under these conditions, the LLM prediction and its confidence score replace the pattern-based result. This selective integration preserves the overall reliability of the pattern-based approach while leveraging the LLM's capability to identify high-risk scenarios with sufficient confidence. Experimental results demonstrate that this hybrid method improves accuracy for high-risk cases while maintaining performance for other risk categories.

Section 4. Evaluation Dataset and Experimental Details

4.1 Evaluation Dataset Construction

Diagnostic Tasks To comprehensively evaluate LLM-based battery failure diagnosis capabilities, two distinct tasks are defined:

Task 1: Failure Cause and Mechanism Diagnosis (FCFM): Given battery material information and experimental data including cycling conditions, observed failure phenomena, and performance metrics as input, the goal is to identify the underlying failure causes and mechanisms. This task requires understanding the causal relationships between failure manifestations and their root causes.

Task 2: Failure Detection Method Recommendation (FDM): Given battery material information and experimental data including cycling conditions, observed failure phenomena, and performance metrics as input, the goal is to recommend appropriate detection methods for identifying and analyzing failures.

Evaluation Dataset Based on the BF-KG, a comprehensive evaluation benchmark was established, comprising two strictly separated components tailored to the diagnostic tasks: a test set for evaluation and a corresponding case base serving as the retrievable knowledge source.

Test Set: To support these diagnostic tasks, a test set was constructed from the BF-KG through a rigorous filtering process:

- **Structural Completeness**: Each case must simultaneously contain all three core node types: Failure Form, Failure Mechanism, and Failure Cause
- **Semantic Uniqueness**: Only one instance per node type is retained within each case to ensure unambiguous causal relationships
- **High-Frequency Coverage**: Cases are filtered to ensure they contain failure types from the top 30 most frequent labels, maintaining dataset representativeness
- **Ground Truth Isolation**: For each diagnostic task, the target answers (failure causes and mechanisms for FCFM, detection methods for FDM) are systematically removed from the cases and preserved as gold standards
- **Information Masking**: For the FCFM diagnostic task, failure detection methods are masked to prevent the model from inferring answers based on detection techniques rather than causal reasoning

Following this construction method, a comprehensive test set comprising 587 rigorously validated cases was established, with 327 cases allocated to the FCFM task and 260 cases to the FDM task.

Case Base: The remaining cases from BF-KG after test data extraction, serving as the retrievable knowledge source for LLMs during diagnosis. In the experiments, this case base acts as the BF-KG accessible to the model, with the extracted test data held out for evaluation only.

Label Standardization

To address label variations from LLM-based extraction, expressions referring to the identical underlying failure concept were unified under a single, consistent label through expert consultation. The final label set is as follows: **Failure Causes (15 labels):**

- Aging
- Electrolyte Decomposition
- High Current Density
- High Temperature
- High Voltage Operation
- Low Temperature
- Mechanical Stress

- Overcharging
- Overdischarge
- Repeated Cycling
- SEI Formation
- Short Circuit
- Side Reactions
- Unstable SEI
- Volume Change

Failure Mechanisms (20 labels):

- Active Material Loss
- Aging
- Degradation
- Dendrite Growth
- Electrolyte Decomposition
- Exothermic Reactions
- Gas Evolution
- Internal Short Circuit
- Lithium Plating
- Mechanical Stress
- Overheating
- Phase Transition
- Polysulfide Shuttle
- SEI Decomposition
- SEI Growth
- Side Reactions
- Structural Degradation
- Thermal Runaway
- Transition Metal Dissolution
- Volume Change

Failure Detection Methods (15 labels):

- SEM
- EIS
- Voltage Monitoring
- Temperature Monitoring

- XRD
- XPS
- Cyclic Voltammetry
- Particle Filter
- Galvanostatic Cycling
- TEM
- Coulomb Counting
- Raman Spectroscopy
- Capacity Measurement
- State Estimation Algorithm
- FTIR

4.2 Experiment Details

In the main experiments, we evaluated the performance gains of the latest mainstream LLMs (DeepSeek-V3.2 [9], Qwen3-Max [10], GPT-5 [11], and LLaMA3.1-8B [12]) when augmented by our proposed BattFailScholar framework. Performance was measured using Accuracy at Top-1, Top-3, and Top-5 to assess both precise diagnostic capability and effectiveness in narrowing down candidate options.

In the experimental setup, to minimize the randomness introduced by large language models, the temperature parameter was set to 0 for all models. However, as the results still exhibited some variability, the final reported results were obtained by averaging multiple independent runs. The models DeepSeek-V3.2, Qwen3-Max, and GPT-5 were accessed via their respective APIs, with DeepSeek-V3.2 specifically using the DeepSeek-V3.2-Terminus version. The LLaMA3.1-8B model was run locally on two NVIDIA GeForce RTX 4090 GPUs.

Since the BattFailScholar framework integrated with DeepSeek-V3.2 demonstrated the best overall performance in the experiments, DeepSeek-V3.2 was selected as the default backbone LLM for the BattFailScholar framework in all subsequent experiments.

4.3 Per-Failure Type Diagnostic Performance

Figure 3-6 presents the per-failure type diagnostic performance comparison before and after applying BattFailScholar.

For failure mechanism diagnosis, before augmentation, 9 out of 19 categories had zero Top-1 accuracy. After applying the framework, zero-accuracy categories were reduced to only 2, with broad improvements across most mechanisms. From the Top-5 perspective, all failure mechanism categories achieved accuracy above 50 percent after augmentation, with 16 out of 19 categories exceeding 70 percent, compared to only 11 before enhancement. These results demonstrate the framework's ability to substantially boost diagnostic reliability across diverse failure types.

For failure cause diagnosis, the task initially exhibited severe sample imbalance, with 5 out of 15 categories having zero Top-1 accuracy and only 4 categories exceeding 50 percent accuracy. After applying BattFailScholar, critical low-performing categories such as High Temperature and Overcharging showed substantial gains. From the Top-5 perspective, all failure cause categories achieved accuracy above 50 percent after augmentation, with 12 out of 15 categories exceeding 70 percent compared to only 8 before enhancement. This indicates that while exact match predictions remain challenging for some categories, the framework consistently ranks the true failure cause among top candidates.

Overall, BattFailScholar effectively alleviates sample imbalance, ensuring that for the vast majority of failure types, the true cause or mechanism is consistently ranked among top candidates, thereby providing more reliable and comprehensive diagnostic support.

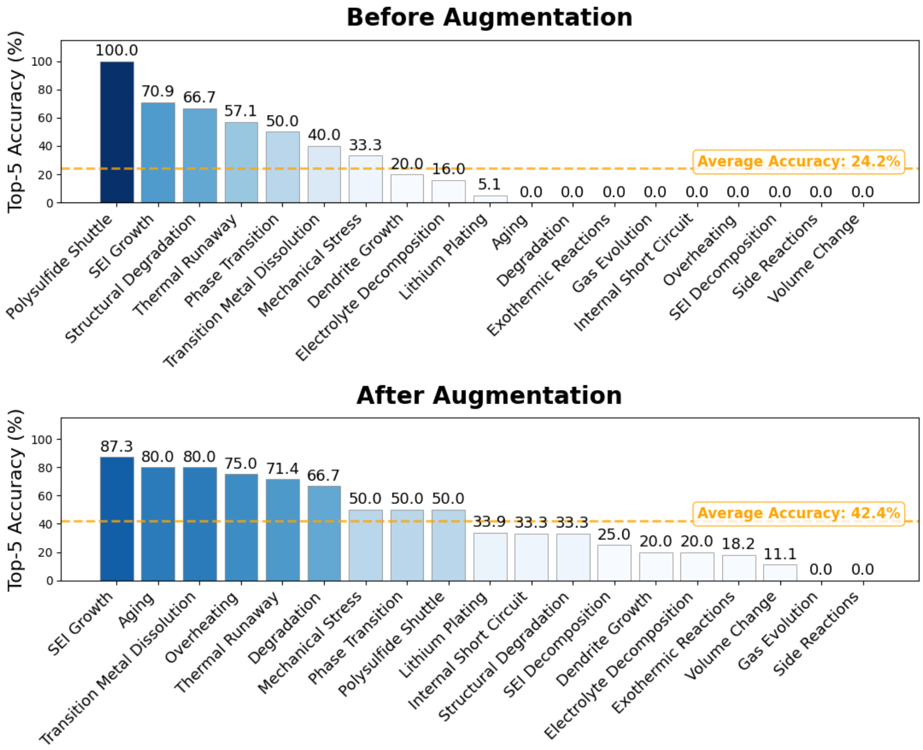

Figure 3: **Per-Failure Mechanism Type Diagnostic Performance (Top-1)**

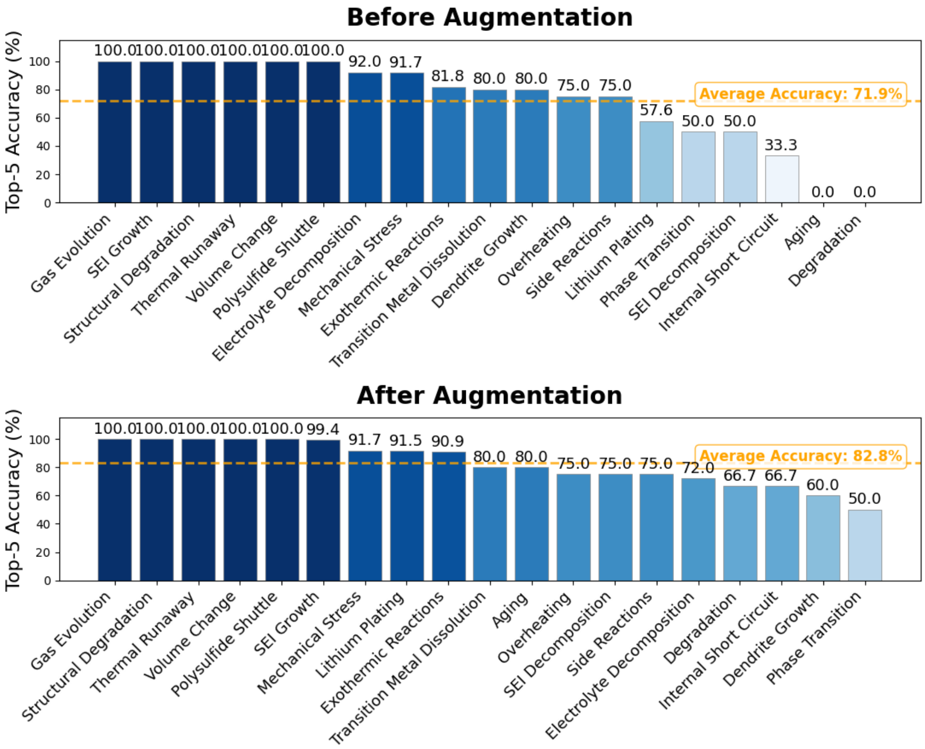

Figure 4: **Per-Failure Mechanism Type Diagnostic Performance (Top-5)**

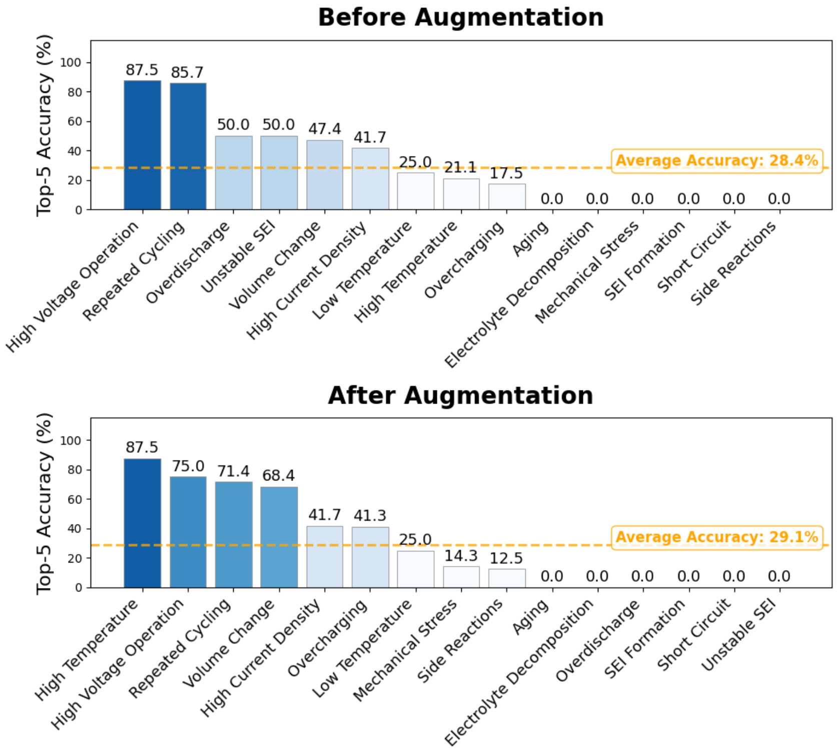

Figure 5: **Per-Failure Cause Type Diagnostic Performance (Top-1)**

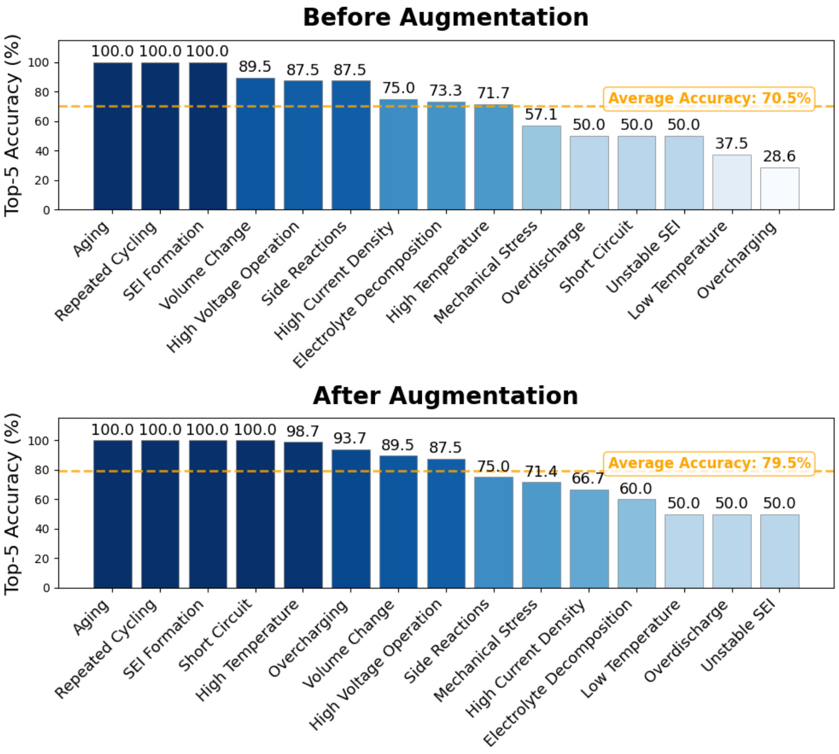

Figure 6: **Per-Failure Cause Type Diagnostic Performance (Top-5)**

4.4 Deep Learning Method Experiment Details

To compare performance with deep learning (DL) methods, Bi-LSTM [13], BERT [14], SciBERT [15], MatBERT[16], GCN [17], and GAT [18] were trained on the constructed battery failure case base and subsequently evaluated on the test dataset to obtain their diagnostic performance. For BERT-based models, a batch size of 16, 5 epochs, and learning rates of 1e-5 to 5e-5 were used. For GNN models, a batch size of 16, 50 epochs with early stopping, and a learning rate of 1e-3 were applied.

The key distinction between these DL approaches and our knowledge-augmented LLM method lies in their learning paradigm: the DL models require training on the entire case base to learn generalized patterns, whereas our method only needs to retrieve a small number of relevant cases (e.g., 10 cases) for in-context learning during inference. Experimental results demonstrate that under this setting, our LLM-based approach not only surpasses the performance of these DL methods but also provides interpretability through the retrieved reference cases and transparent reasoning process.

4.5 Comparative Experiment

We also compared our BattFailScholar framework with representative GraphRAG-based and CBR-based methods. These methods are described as follows:

- **GraphRAG** [19]: Constructs a knowledge graph with hierarchical community summaries, enabling models to answer complex questions requiring a macro-level understanding.
- **LightRAG** [20]: A lightweight evolution of GraphRAG that simplifies hierarchical communities into a dual-layer retrieval mechanism, capturing both local details and global themes at much lower cost.
- **SimGRAG** [21]: Converts queries into structured pattern graphs and searches for semantically and structurally aligned subgraphs, ensuring precise matching.
- **T^2RAG** [22]: Employs iterative triple filling for efficient reasoning, avoiding the overhead of full graph construction and multi-turn retrieval.
- **KG-Agent** [23]: Transforms LLMs into agents with dynamic memory and graph query tools, enabling autonomous step-by-step exploration for multi-hop reasoning.
- **KNN** [24]: As a representative CBR approach, KNN retrieves similar cases and directly uses the retrieved results as final answers. Here, we take the most frequent result among the top-10 retrieved cases as the final prediction.
- **KNN** [24] **+ Our FFR**: This variant replaces the conventional semantic similarity-based retrieval in KNN with our proposed Failure Feature-Aware Retrieval (FFR) algorithm, while keeping the subsequent voting mechanism unchanged. This ablation helps validate the effectiveness of our failure-aware retrieval strategy.

The complete comparative results are presented in Figure 7. In contrast to these approaches, our BattFailScholar framework offers two key advantages: (1) Unlike GraphRAG variants that rely on graph structures and community summaries, our FFR algorithm directly leverages case-level failure-specific features for more targeted retrieval in battery failure scenarios; (2) Unlike traditional CBR methods that simply vote on retrieved results, our framework combines failure-aware retrieval with LLM-based reasoning, enabling deeper causal understanding rather than surface-level pattern matching. This failure-domain specialization makes BattFailScholar particularly suited for battery failure diagnosis tasks.

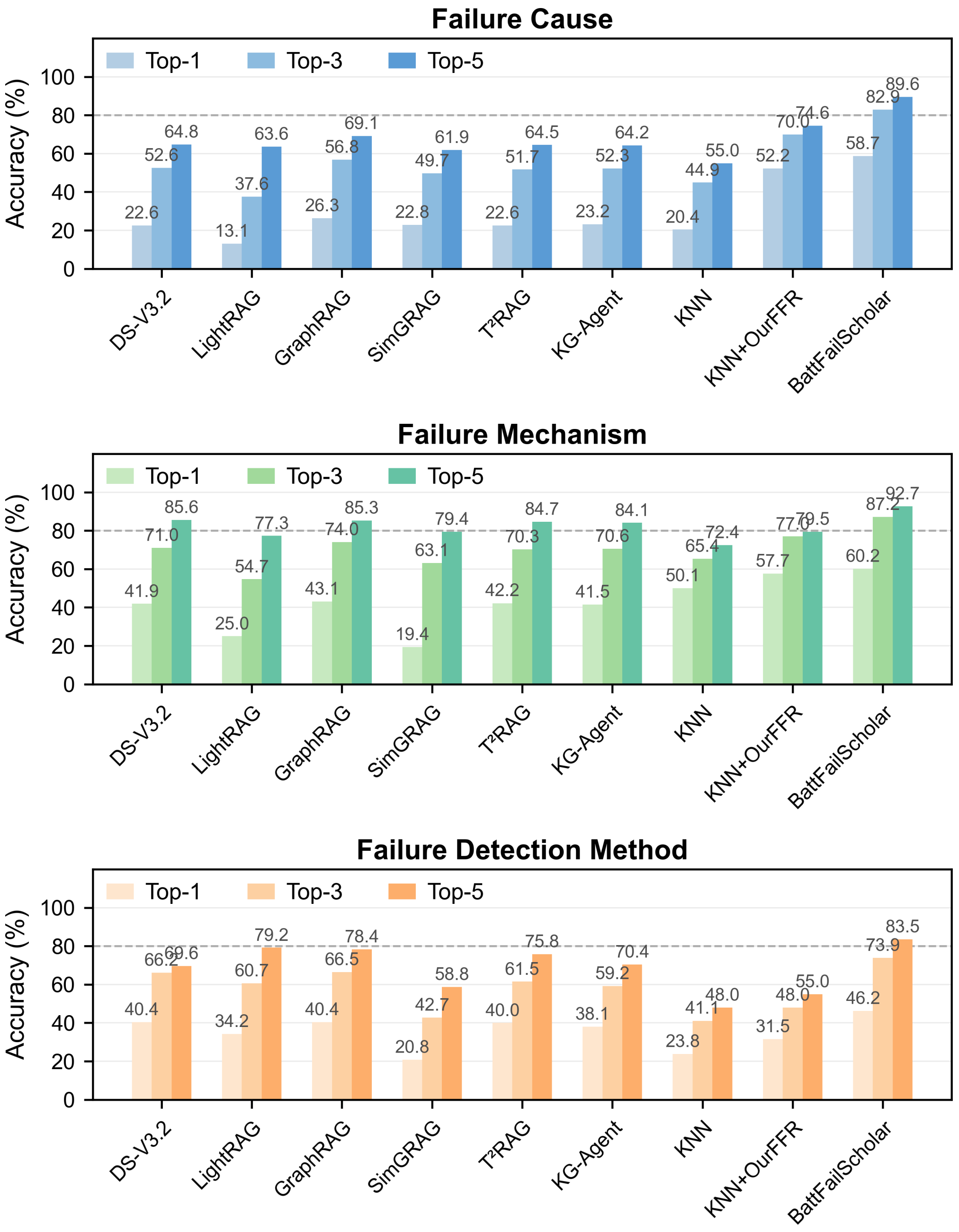

Figure 7: **Performance comparison of BattFailScholar with GraphRAG-based and CBR-based methods on battery failure diagnosis tasks.**

4.6 Ablation Experiment

The following variant methods were constructed and evaluated to analyze the contribution of each component in the BattFailScholar (BFS) framework, as summarized in Table 9:

- **DeepSeek-V3.2**: The baseline method using only the base LLM with task instruction and candidate labels, without any knowledge augmentation.
- **BFS**: The complete proposed BattFailScholar framework, integrating the failure-aware case graph retrieval algorithm with the base LLM for diagnosis.
- **DeepSeek-V3.2 with CoT in inference**: The baseline LLM enhanced with Chain-of-Thought (CoT)[25] prompting during inference, testing whether explicit reasoning steps alone can improve diagnostic accuracy without knowledge augmentation.
- **BFS with CoT in inference**: The complete framework further enhanced with Chain-of-Thought prompting during inference, examining whether combining knowledge augmentation with explicit reasoning provides additional performance gains.
- **BFS with random case retrieval**: A variant where the failure-aware retrieval algorithm is replaced by random selection of cases from the BF-KG, maintaining the same number of retrieved cases but removing semantic relevance.
- **BFS with whole-case retrieval**: A variant where case similarity is computed at the entire case graph level rather than using fine-grained triplet-level matching, testing the importance of granular semantic alignment.
- **BFS with paper replacing KG in inference**: A variant where structured case graphs in the prompt are replaced by the original paper text, testing the advantage of case graph representation over full text for supporting LLM diagnosis.

Table 9: Ablation Study Variants of the BattFailScholar (BFS) Framework

Variant Method	Retrieval Phase	LLM Inference Phase
DeepSeek-V3.2 (Baseline)	– No retrieval	– No cases
BFS (Ours)	+ Failure-aware case graph retrieval	+ Structured case graphs
DeepSeek-V3.2 with CoT in inference	– No retrieval	+ Chain-of-Thought
BFS with CoT in inference	+ Failure-aware case graph retrieval	+ Structured case graphs<br>+ Chain-of-Thought
BFS with random case retrieval	– Failure-aware case graph retrieval<br>+ Random retrieval	+ Structured case graphs
BFS with whole-case retrieval	– Failure-aware case graph retrieval<br>+ Entire case-level similarity	+ Structured case graphs
BFS with paper replacing KG	+ Failure-aware case graph retrieval	– Structured case graphs<br>+ Original paper text

These ablation variants systematically isolate the effects of knowledge augmentation, fine-grained failure-aware case graph retrieval, and reasoning enhancement via CoT, providing comprehensive insights into the framework's key success factors.

4.7 Cross-Source Generalization Evaluation

To further validate the generalization capability of BattFailScholar, an additional test set was constructed using failure literature from a different source (CNKI). While the original test set and case base share inherent

homogeneity from the same source (Google Scholar), this evaluation assesses whether the method maintains effectiveness on cross-source data.

Test Set Construction A collection of 220 academic publications was obtained from CNKI using ”lithium-ion battery failure” keywords. Following the same extraction methodology described in Section 1.3 and test set construction criteria in Section 4.1, with the relaxed requirement of containing failure form paired with either failure cause or failure mechanism, 29 valid test cases were obtained: 15 with failure cause information and 21 with failure mechanism information. Detection method evaluation was excluded due to insufficient data.

Evaluation and Results Using the original case base (Google Scholar-derived) as the retrieval source without any modification, BattFailScholar was evaluated on this cross-source test set. Results demonstrate that performance improvements were still achieved despite the domain shift between literature sources, validating the cross-source generalization capability of the proposed approach. This confirms that the knowledge graph representation and retrieval mechanism are robust to variations in data origin.

4.8 Laboratory Data Evaluation

To further validate the practical applicability of BattFailScholar on real experimental data, experiments were conducted using the thermal runaway battery failure dataset [26] for trigger mechanism prediction (i.e., inferring the cause that led to thermal runaway).

Data Preparation For each experimental record, trigger process parameters that would directly reveal the answer were removed from the input data. Battery specifications, initial conditions, and failure results were retained as valid input features. The dataset was randomly split into training, validation, and test sets (8:1:1).

Deep Learning Baseline A BERT-based classifier was implemented for comparison. Each experimental record was converted into key-value text format and encoded using MatBERT[16]. The model was trained for 10 epochs with batch size 16 and learning rate 2e-5.

LLM-Based Evaluation Since this thermal runaway dataset contains specialized experimental parameters not covered in the literature-derived BF-KG, the training set was used as the retrieval source to ensure fair comparison with deep learning methods trained on the same data. Two variants were evaluated: base LLM (direct prediction) and KAG-enhanced LLM (with retrieved cases).

4.9 Failure Cluster Visualization of BF-KG

The construction quality of the BF-KG provides the foundational structure for organizing battery failure knowledge, while the effectiveness of the case vectorization method is critical for leveraging this structure in downstream retrieval and diagnostic tasks. To quantitatively evaluate whether the BF-KG meaningfully organizes knowledge and whether our vectorization method accurately captures semantic similarities, an embedding-based clustering analysis was conducted. This analysis verifies that cases sharing similar failure attributes are positioned closer in the vector space, thereby assessing the reliability of the entire framework for representing and reasoning about battery failure knowledge.

Specifically, the entire BF-KG was vectorized and clustered at the paper-case level using the triple embedding method from the failure feature-aware retrieval module, which encodes semantic relationships through a sentence transformer. The resulting case vectors were projected into a two-dimensional space using t-SNE for visualization. The analysis specifically focused on the top 10 most frequent failure types within each category, with distinct coloring applied according to failure type labels to evaluate cluster coherence and separability.

The t-SNE visualization results revealed clearly separated clusters corresponding to distinct failure types, as shown in Figures 8-10. This demonstrates that the case vectorization method successfully maps semantically similar cases from the BF-KG into proximate regions of the vector space. All three major categories of failure form, mechanism, and cause formed cohesive groups with minimal overlap, validating both the structural quality of the knowledge graph and the discriminative power of the case vectorization method. These well-defined clusters confirm that our representation approach effectively supports downstream tasks such as failure case retrieval and diagnosis.

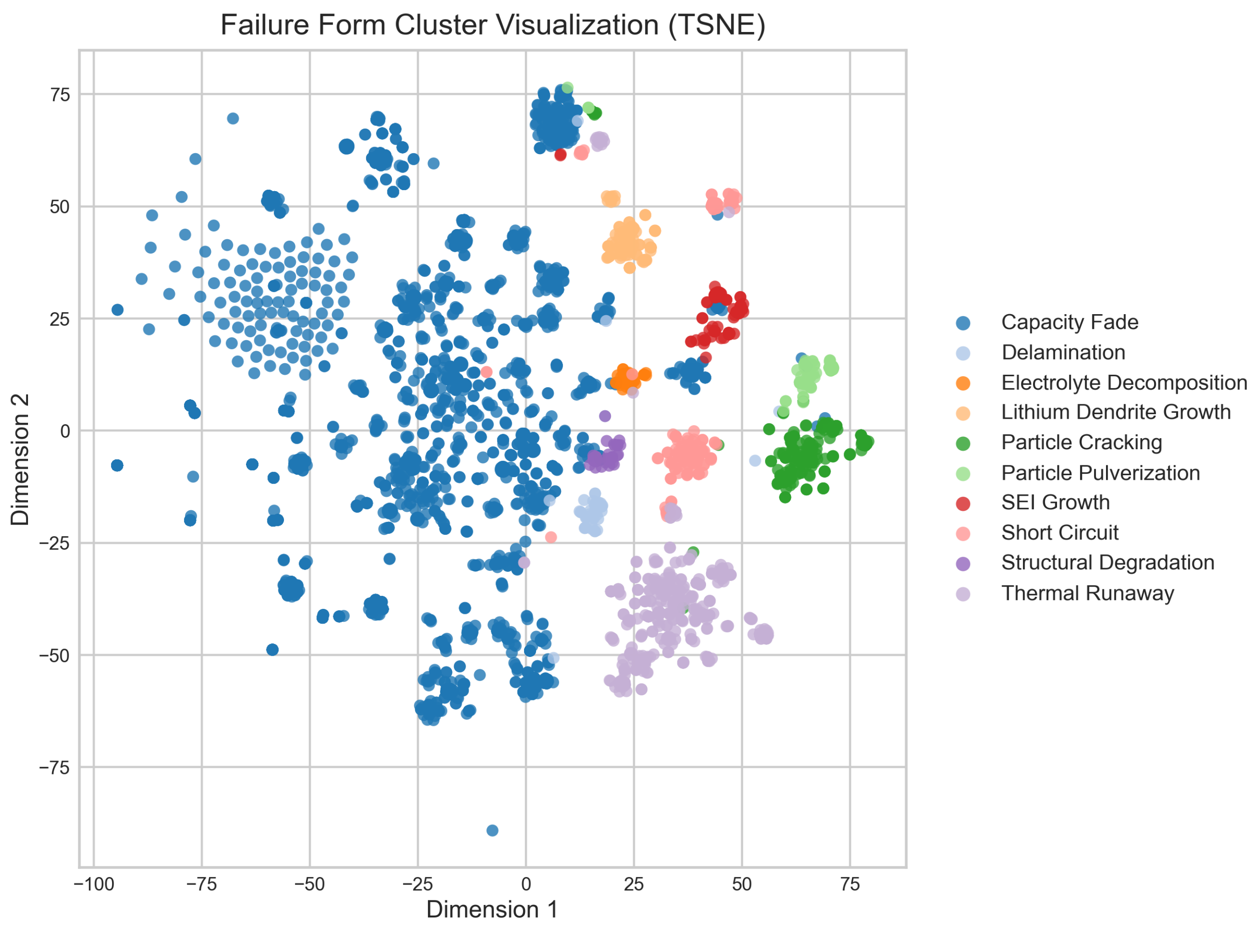

Figure 8: **BF-KG Case Clustering Visualization by Failure Form (Top-10)**. The clustering results reveal several important patterns. The overwhelming dominance of ”Capacity Fade” across cases indicates that this failure mode represents a common performance endpoint with diverse underlying causes, making it difficult to infer specific failure mechanisms or root causes from this category alone. In contrast, well-defined and distinct clusters such as ”Lithium Dendrite Growth,” ”Short Circuit,” and ”Thermal Runaway” represent failure modes with more specific and identifiable characteristics. Additionally, the close proximity between ”Particle Cracking” and ”Particle Pulverization” clusters reflects their shared origin in mechanical degradation processes, suggesting that the vectorization method successfully captures their semantic and mechanistic relatedness.

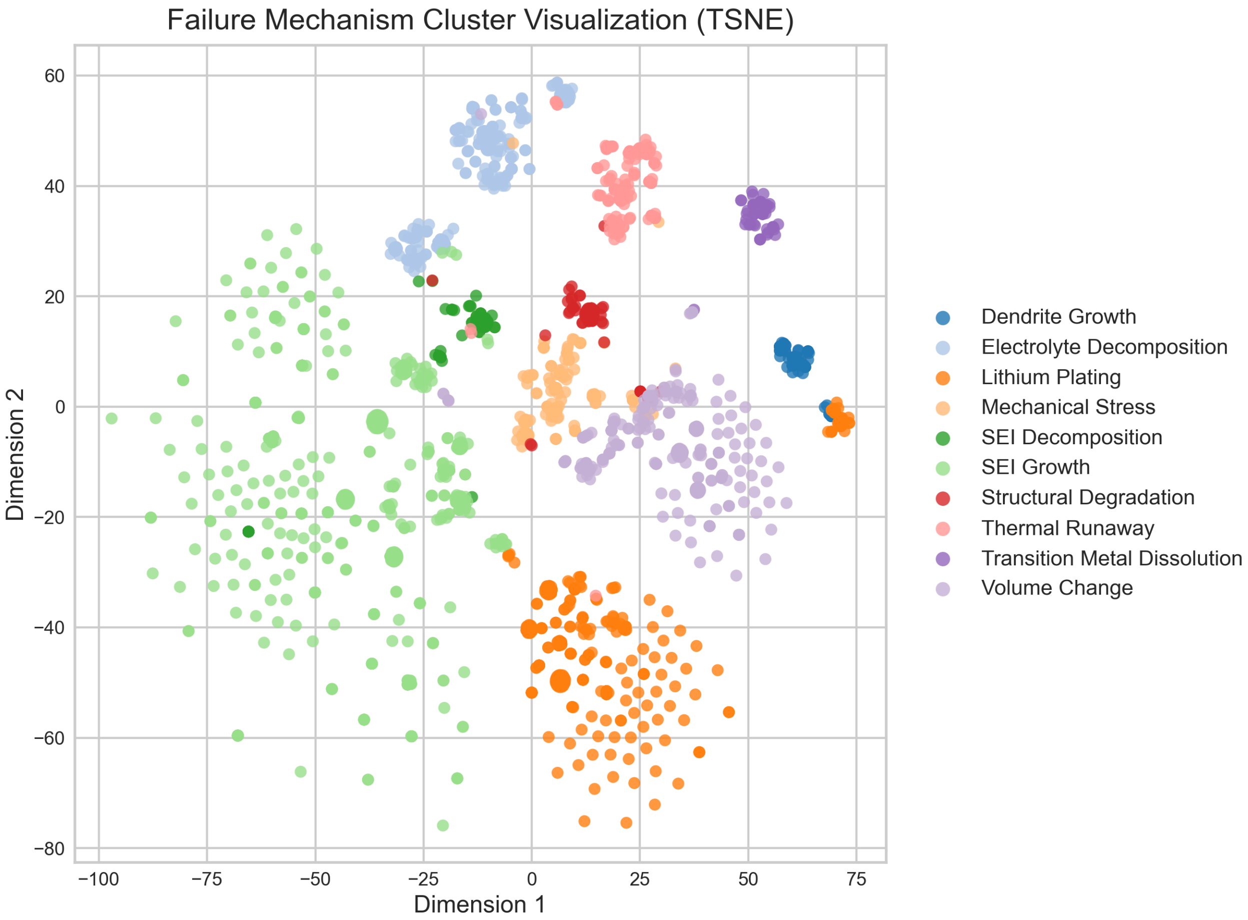

Figure 9: **BF-KG Case Clustering Visualization by Failure Mechanism (Top-10)**.The clustering of failure mechanisms demonstrates strong separability across different categories. The prevalence of ”SEI Growth” as the dominant mechanism aligns with its recognized role as a fundamental degradation process in most battery systems. The observed proximity between ”SEI Decomposition” and ”SEI Growth” reflects their shared chemical nature within the interphase evolution process, while the clustering of ”Mechanical Stress” near ”Volume Change” corresponds well with their coupled physical effects during electrode cycling. Notably, sub-cluster structures within ”Electrolyte Decomposition” and ”Lithium Plating” suggest that while these mechanisms form coherent categories, they may manifest through distinct pathways or under different operational conditions, highlighting the multi-faceted nature of failure mechanisms.

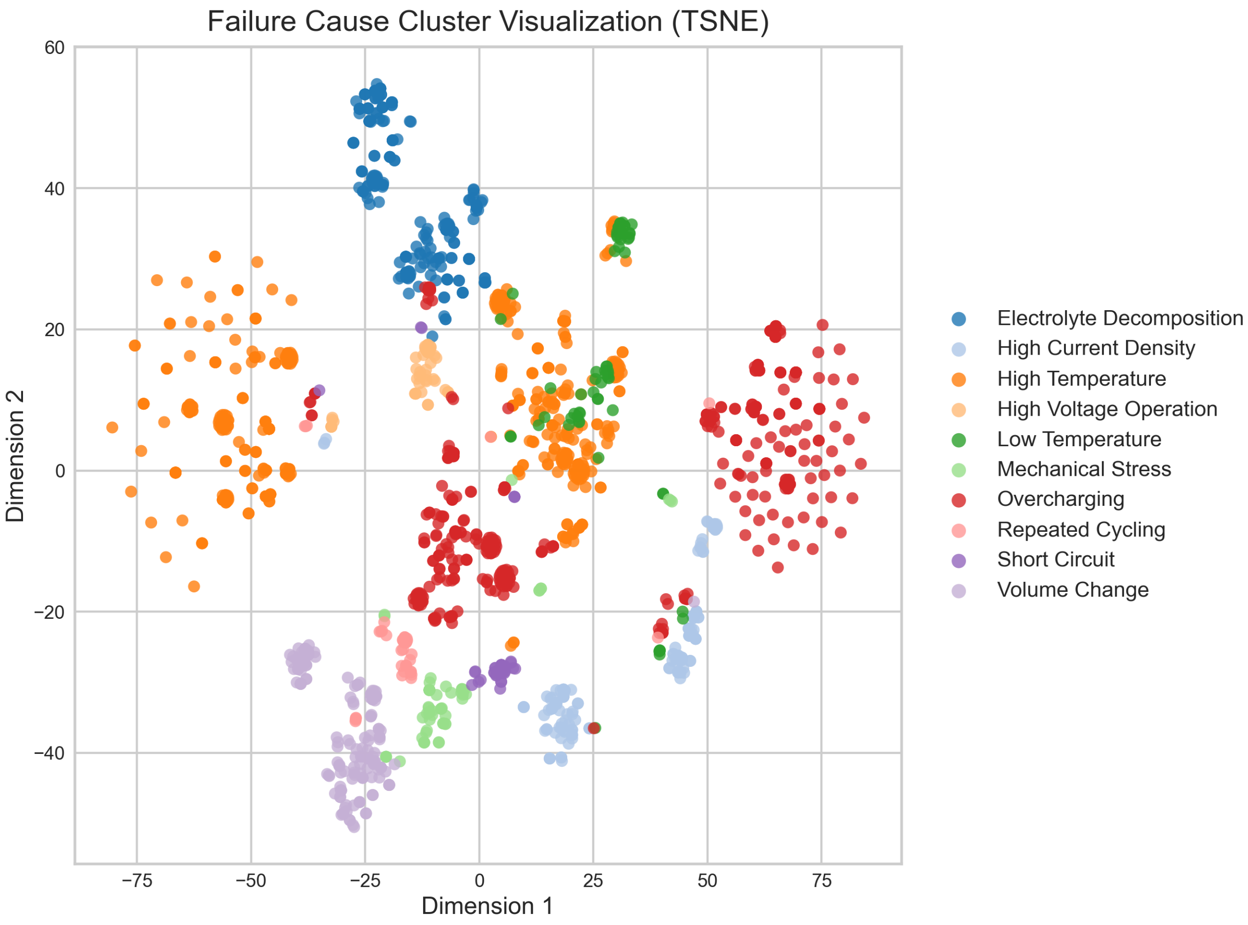

Figure 10: **BF-KG Case Clustering Visualization by Failure Cause (Top-10)**. The clustering of failure causes exhibits clear separation overall, though with slightly more distributional dispersion compared to failure forms and mechanisms. The emergence of sub-clusters within categories such as ”Overcharging” and ”High Temperature” indicates that a single failure cause may encompass multiple distinct scenarios or severity levels. Particularly noteworthy is the significant overlap observed between the ”High Temperature” and ”Low Temperature” clusters, suggesting that despite representing opposite thermal conditions, they may result in comparable symptom manifestations or operate through similar underlying pathways that our vectorization method captures as proximate in the embedding space.

4.10 Case Study

To demonstrate the practical application of our diagnostic framework, a case study is presented comparing the baseline and enhanced diagnostic approaches:

Basic LLM-based Diagnosis: Battery failure diagnosis using the DeepSeek-V3.2 foundation model without specialized enhancement or domain knowledge integration. The input prompts and corresponding generated results are shown below:

Input Prompt:

Basic LLM-based Battery Failure Diagnosis Prompt

```
Battery Failure Analysis

The BatteryCase "Retired_EV_Battery" --hasMaterialComponent--> Cathode Material "Li-ion".
The BatteryCase "Retired_EV_Battery" --hasStructureAttribute--> Cell Level Information "80%
     SOH".
The BatteryCase "Retired_EV_Battery" --undergoesProcess--> ProcessStep "Calendar_Aging".
The BatteryCase "Retired_EV_Battery" --undergoesProcess--> ProcessStep "Cycle_Aging".
The ProcessStep "Calendar_Aging" --hasCondition--> Temperature "High".
The ProcessStep "Cycle_Aging" --hasCondition--> DOD "30%".
The ProcessStep "Calendar_Aging" --followedBy--> ProcessStep "Cycle_Aging".
The ProcessStep "Cycle_Aging" --hasObservation--> Observation "Capacity_Degradation".
The Observation "Capacity_Degradation" --hasSignal--> Electrical Signal "Capacity 62.29%".
The Observation "Capacity_Degradation" --hasPerformanceMetric--> SOH "80% to 62.29%".
The Observation "Capacity_Degradation" --hasFailureForm--> Failure Form "Capacity Fade".
The Observation "Capacity_Degradation" --occursAt--> CyclePoint "1_Year".
The BatteryCase "Retired_EV_Battery" --hasMonitoringData--> Monitoring "SOH_Distribution".
The BatteryCase "Retired_EV_Battery" --inScenario--> Usage Scenario "Grid_Storage".
The ProcessStep "Cycle_Aging" --hasObservation--> Observation "Module_Capacity".
The Observation "Module_Capacity" --hasSignal--> Electrical Signal "Capacity 40 MWh".
The Observation "Module_Capacity" --hasPerformanceMetric--> Reliability "Improved".
The Observation "Module_Capacity" --occursAt--> CyclePoint "Initial_Deployment".

Candidate Failure Causes:
Aging, Electrolyte Decomposition, High Current Density, High Temperature, High Voltage
     Operation, Low Temperature, Mechanical Stress, Overcharging, Overdischarge, Repeated
     Cycling, SEI Formation, Short Circuit, Side Reactions, Unstable SEI, Volume Change

Candidate Failure Mechanisms:
Active Material Loss, Aging, Degradation, Dendrite Growth, Electrolyte Decomposition,
     Exothermic Reactions, Gas Evolution, Internal Short Circuit, Lithium Plating, Mechanical
      Stress, Overheating, Phase Transition, Polysulfide Shuttle, SEI Decomposition, SEI
     Growth, Side Reactions, Structural Degradation, Thermal Runaway, Transition Metal
     Dissolution, Volume Change

Based on the battery structure and observation data provided above, please infer the top-1
     most likely Failure Causes and Failure Mechanisms of the system. Select exactly 1 items
     from each candidate list.

Answer in the following JSON format:
{
  "Failure Cause": ["...", "...", "..."],
  "Failure Mechanism": ["...", "...", "..."]
}
```

Generated Output:

Generated Output


```
{
"Failure Cause": ["Aging"],
"Failure Mechanism": ["Active Material Loss"]
}
```


BattFailScholar-Enhanced Diagnosis: Battery failure diagnosis implemented through the proposed BattFailScholar framework, which augments the DeepSeek-V3.2 model with integrated failure case knowledge from BF-KG:

Input Prompt:

BattFailScholar-Enhanced Diagnosis Prompt

```
Battery Failure Analysis

The BatteryCase "Retired_EV_Battery" --hasMaterialComponent--> Cathode Material "Li-ion".
The BatteryCase "Retired_EV_Battery" --hasStructureAttribute--> Cell Level Information "80%
     SOH".
The BatteryCase "Retired_EV_Battery" --undergoesProcess--> ProcessStep "Calendar_Aging".
The BatteryCase "Retired_EV_Battery" --undergoesProcess--> ProcessStep "Cycle_Aging".
The ProcessStep "Calendar_Aging" --hasCondition--> Temperature "High".
The ProcessStep "Cycle_Aging" --hasCondition--> DOD "30%".
The ProcessStep "Calendar_Aging" --followedBy--> ProcessStep "Cycle_Aging".
The ProcessStep "Cycle_Aging" --hasObservation--> Observation "Capacity_Degradation".
The Observation "Capacity_Degradation" --hasSignal--> Electrical Signal "Capacity 62.29%".
The Observation "Capacity_Degradation" --hasPerformanceMetric--> SOH "80% to 62.29%".
The Observation "Capacity_Degradation" --hasFailureForm--> Failure Form "Capacity Fade".
The Observation "Capacity_Degradation" --occursAt--> CyclePoint "1_Year".
The BatteryCase "Retired_EV_Battery" --hasMonitoringData--> Monitoring "SOH_Distribution".
The BatteryCase "Retired_EV_Battery" --inScenario--> Usage Scenario "Grid_Storage".
The ProcessStep "Cycle_Aging" --hasObservation--> Observation "Module_Capacity".
The Observation "Module_Capacity" --hasSignal--> Electrical Signal "Capacity 40 MWh".
The Observation "Module_Capacity" --hasPerformanceMetric--> Reliability "Improved".
The Observation "Module_Capacity" --occursAt--> CyclePoint "Initial_Deployment".


# Related Historical Cases

## Case 1:
The BatteryCase "EV_Battery" --hasMaterialComponent--> Anode Material "Graphite".
The BatteryCase "EV_Battery" --hasMaterialComponent--> Cathode Material "NMC".
The BatteryCase "EV_Battery" --hasMaterialComponent--> Cathode Material "NCA".
The BatteryCase "EV_Battery" --hasMaterialComponent--> Cathode Material "LFP".
The BatteryCase "EV_Battery" --hasStructureAttribute--> Porosity "Inhomogeneous".
The BatteryCase "EV_Battery" --hasStructureAttribute--> Thickness "Variable".
The BatteryCase "EV_Battery" --undergoesProcess--> ProcessStep "Calendrical_Aging".
The BatteryCase "EV_Battery" --undergoesProcess--> ProcessStep "Cycle_Aging".
The ProcessStep "Calendrical_Aging" --hasCondition--> Temperature "High".
The ProcessStep "Calendrical_Aging" --hasCondition--> State Of Charge "High".
The ProcessStep "Cycle_Aging" --hasCondition--> Current Rate "High".
The ProcessStep "Cycle_Aging" --hasCondition--> Depth Of Discharge "High".
The ProcessStep "Calendrical_Aging" --followedBy--> ProcessStep "Cycle_Aging".
The ProcessStep "Cycle_Aging" --hasObservation--> Observation "Capacity_Fade".
The ProcessStep "Cycle_Aging" --hasObservation--> Observation "Resistance_Increase".
The Observation "Capacity_Fade" --hasSignal--> Electrical Signal "Capacity 80%".
The Observation "Capacity_Fade" --hasPerformanceMetric--> SOH "80%".
The Observation "Resistance_Increase" --hasSignal--> Electrical Signal "Resistance 120%".
The Observation "Capacity_Fade" --hasFailureForm--> Failure Form "Non-linear Aging".
The Failure Form "Non-linear Aging" --hasFailureMechanism--> Failure Mechanism "Lithium
     Plating".
The Failure Mechanism "Lithium Plating" --hasFailureCause--> Failure Cause "High Charging
     Rate".
The Failure Mechanism "Lithium Plating" --hasFailureCause--> Failure Cause "Low Temperature".
The Failure Mechanism "Lithium Plating" --hasFailureCause--> Failure Cause "High DoD".
The Observation "Capacity_Fade" --occursAt--> CyclePoint "EOL".
The BatteryCase "EV_Battery" --hasMonitoringData--> Monitoring "sBMS".
The Monitoring "sBMS" --detectedBy--> Detection Method "Voltage Monitoring".
The Monitoring "sBMS" --detectedBy--> Detection Method "Temperature Monitoring".
The BatteryCase "EV_Battery" --inScenario--> Usage Scenario "Second Life Storage".

## Case 2:
The BatteryCase "Li-ion EV Battery" --hasMaterialComponent--> Anode Material "Graphite".
The BatteryCase "Li-ion EV Battery" --hasMaterialComponent--> Cathode Material "LFP (LiFePO4)
     ".
The BatteryCase "Li-ion EV Battery" --hasMaterialComponent--> Cathode Material "NMC (
     LiNixCoyMn1-x-yO2)".
The BatteryCase "Li-ion EV Battery" --hasMaterialComponent--> Electrolyte "Organic Polymer
     Solution".
The BatteryCase "Li-ion EV Battery" --undergoesProcess--> ProcessStep "Calendar Aging".
The BatteryCase "Li-ion EV Battery" --undergoesProcess--> ProcessStep "Cycle Aging".
The ProcessStep "Cycle Aging" --followedBy--> ProcessStep "Calendar Aging".
The ProcessStep "Cycle Aging" --hasObservation--> Observation "Capacity Fade".
The ProcessStep "Cycle Aging" --hasObservation--> Observation "Power Reduction".
The Observation "Capacity Fade" --hasSignal--> Electrical Signal "Capacity 70%".
The Observation "Capacity Fade" --hasPerformanceMetric--> Capacity Retention "30% loss".
The Observation "Capacity Fade" --hasFailureForm--> Failure Form "Active Material Loss".
The Observation "Capacity Fade" --hasFailureMechanism--> Failure Mechanism "SEI Growth".
The Observation "Capacity Fade" --hasFailureMechanism--> Failure Mechanism "Lithium Plating".
The Failure Mechanism "SEI Growth" --hasFailureCause--> Failure Cause "High Temperature".
The Failure Mechanism "Lithium Plating" --hasFailureCause--> Failure Cause "Low Temperature".
The Observation "Power Reduction" --hasSignal--> Electrical Signal "Increased Impedance".
The Observation "Power Reduction" --hasFailureForm--> Failure Form "Transport Barriers".
```

```
The Observation "Power Reduction" --hasFailureMechanism--> Failure Mechanism "CEI Formation".
The Observation "Capacity Fade" --occursAt--> CyclePoint "160,000 km".
The Observation "Capacity Fade" --occursAt--> CyclePoint "8 years".
The BatteryCase "Li-ion EV Battery" --hasMonitoringData--> Monitoring "BMS Data".
The BatteryCase "Li-ion EV Battery" --inScenario--> Usage Scenario "Urban Driving".
The ProcessStep "Cycle Aging" --hasCondition--> Condition "High C-rate".
The ProcessStep "Cycle Aging" --hasCondition--> Condition "Deep DOD".
The ProcessStep "Calendar Aging" --hasCondition--> Condition "Ambient Temperature".

## Case 3:
The BatteryCase "Lithium-ion EV Battery" --hasMaterialComponent--> Cathode Material "Lithium-
     based".
The BatteryCase "Lithium-ion EV Battery" --hasMaterialComponent--> Anode Material "Graphite".
The BatteryCase "Lithium-ion EV Battery" --hasMaterialComponent--> Electrolyte "Organic
     solvent with lithium salts".
The BatteryCase "Lithium-ion EV Battery" --undergoesProcess--> ProcessStep "Calendar Aging".
The BatteryCase "Lithium-ion EV Battery" --undergoesProcess--> ProcessStep "Cycle Aging".
The ProcessStep "Calendar Aging" --followedBy--> ProcessStep "Cycle Aging".
The ProcessStep "Cycle Aging" --hasObservation--> Observation "Capacity Fade".
The ProcessStep "Cycle Aging" --hasObservation--> Observation "Impedance Increase".
The Observation "Capacity Fade" --hasSignal--> Electrical Signal "Capacity Loss".
The Observation "Impedance Increase" --hasSignal--> Electrical Signal "Resistance Growth".
The Observation "Capacity Fade" --hasPerformanceMetric--> SOH "80%".
The Observation "Capacity Fade" --occursAt--> CyclePoint "EOL".
The Observation "Capacity Fade" --hasFailureForm--> Failure Form "Capacity Fade".
The Observation "Impedance Increase" --hasFailureForm--> Failure Form "Resistance Growth".
The Failure Form "Capacity Fade" --hasFailureMechanism--> Failure Mechanism "Lithium-ion Loss
     ".
The Failure Form "Resistance Growth" --hasFailureMechanism--> Failure Mechanism "SEI Growth".
The Failure Mechanism "Lithium-ion Loss" --hasFailureCause--> Failure Cause "Overcharging".
The Failure Mechanism "SEI Growth" --hasFailureCause--> Failure Cause "High Temperature".
The BatteryCase "Lithium-ion EV Battery" --hasMonitoringData--> Monitoring "Coulomb Counting
     ".
The BatteryCase "Lithium-ion EV Battery" --hasMonitoringData--> Monitoring "EKF".
The ProcessStep "Cycle Aging" --hasCondition--> Condition "High Temperature".
The ProcessStep "Cycle Aging" --hasCondition--> Condition "High SOC".
The ProcessStep "Cycle Aging" --hasCondition--> Condition "High Charge Current".
The Observation "Capacity Fade" --detectedBy--> Detection Method "Spectroscopy".
The Observation "Impedance Increase" --detectedBy--> Detection Method "Electrochemical
     Impedance Spectroscopy".
The BatteryCase "Lithium-ion EV Battery" --inScenario--> Usage Scenario "Electric Vehicle".

## Case 4:
The BatteryCase "Li-ion" --hasMaterialComponent--> Anode Material "Graphite".
The BatteryCase "Li-ion" --hasMaterialComponent--> Cathode Material "Lithium Metal Oxides".
The BatteryCase "Li-ion" --hasMaterialComponent--> Electrolyte "Lithium Salt Solution".
The BatteryCase "Li-ion" --undergoesProcess--> ProcessStep "Cycling Aging".
The BatteryCase "Li-ion" --undergoesProcess--> ProcessStep "Calendar Aging".
The ProcessStep "Cycling Aging" --followedBy--> ProcessStep "Calendar Aging".
The ProcessStep "Cycling Aging" --hasObservation--> Observation "Capacity Degradation".
The ProcessStep "Cycling Aging" --hasObservation--> Observation "Resistance Increase".
The ProcessStep "Calendar Aging" --hasObservation--> Observation "SEI Formation".
The Observation "Capacity Degradation" --hasSignal--> Electrical Signal "Capacity".
The Observation "Capacity Degradation" --hasPerformanceMetric--> SOH "70%".
The Observation "Resistance Increase" --hasSignal--> Electrical Signal "Internal Resistance".
The Observation "SEI Formation" --hasFailureForm--> Failure Form "Solid Electrolyte
     Interphase".
The Observation "SEI Formation" --hasFailureMechanism--> Failure Mechanism "Electrolyte
     Decomposition".
The Failure Mechanism "Electrolyte Decomposition" --hasFailureCause--> Failure Cause "High
     Temperature Storage".
The Observation "Capacity Degradation" --occursAt--> CyclePoint "500".
The Observation "Resistance Increase" --occursAt--> CyclePoint "500".
The Observation "SEI Formation" --occursAt--> CyclePoint "30 days".
The BatteryCase "Li-ion" --inScenario--> Usage Scenario "Electric Vehicles".
The BatteryCase "Li-ion" --inScenario--> Usage Scenario "Grid Storage".
The BatteryCase "Li-ion" --hasMonitoringData--> Monitoring Data "NASA Battery Dataset".
The Observation "Capacity Degradation" --detectedBy--> Detection Method "Charge Capacity
     Tracking".
The Observation "Resistance Increase" --detectedBy--> Detection Method "Voltage Profile
     Analysis".
The Observation "SEI Formation" --detectedBy--> Detection Method "Differential Thermal
     Voltammetry".

## Case 5:
The BatteryCase "1" --hasMaterialComponent--> Anode Material "Graphite".
The BatteryCase "1" --hasMaterialComponent--> Cathode Material "LiCoO2".
The BatteryCase "1" --hasMaterialComponent--> Electrolyte "Ethyl Carbonate".
The BatteryCase "1" --undergoesProcess--> ProcessStep "Calendar Ageing".
The BatteryCase "1" --undergoesProcess--> ProcessStep "Cycle Ageing".
The ProcessStep "Calendar Ageing" --hasCondition--> Condition "High SOC Storage".
```

```
The ProcessStep "Calendar Ageing" --hasCondition--> Condition "High Temperature".
The ProcessStep "Cycle Ageing" --hasCondition--> Condition "High DSOC".
The ProcessStep "Cycle Ageing" --hasCondition--> Condition "High Voltage".
The ProcessStep "Calendar Ageing" --hasObservation--> Observation "Capacity Fade".
The ProcessStep "Cycle Ageing" --hasObservation--> Observation "Resistance Growth".
The Observation "Capacity Fade" --hasSignal--> Electrical Signal "Capacity".
The Observation "Capacity Fade" --hasPerformanceMetric--> SOH "80%".
The Observation "Resistance Growth" --hasSignal--> Electrical Signal "Impedance".
The Observation "Resistance Growth" --hasPerformanceMetric--> Power "Reduced Max Power".
The Observation "Capacity Fade" --hasFailureForm--> Failure Form "Capacity Fade".
The Observation "Resistance Growth" --hasFailureForm--> Failure Form "Resistance Increase".
The Failure Form "Capacity Fade" --hasFailureMechanism--> Failure Mechanism "SEI Growth".
The Failure Form "Capacity Fade" --hasFailureMechanism--> Failure Mechanism "Lithium Plating
     ".
The Failure Form "Resistance Increase" --hasFailureMechanism--> Failure Mechanism "SEI
     Dissolution".
The Failure Mechanism "SEI Growth" --hasFailureCause--> Failure Cause "High Temperature".
The Failure Mechanism "SEI Growth" --hasFailureCause--> Failure Cause "High SOC".
The Failure Mechanism "Lithium Plating" --hasFailureCause--> Failure Cause "Low Temperature".
The Failure Mechanism "SEI Dissolution" --hasFailureCause--> Failure Cause "High Voltage".
The Observation "Capacity Fade" --occursAt--> CyclePoint "1000".
The Observation "Resistance Growth" --occursAt--> CyclePoint "500".
The ProcessStep "Calendar Ageing" --followedBy--> ProcessStep "Cycle Ageing".
The BatteryCase "1" --hasMonitoringData--> Monitoring "Coulomb Counting".
The BatteryCase "1" --hasMonitoringData--> Monitoring "EIS Measurements".
The BatteryCase "1" --inScenario--> Scenario "Electric Vehicle Use".

.......

Candidate Failure Causes:
Aging, Electrolyte Decomposition, High Current Density, High Temperature, High Voltage
     Operation, Low Temperature, Mechanical Stress, Overcharging, Overdischarge, Repeated
     Cycling, SEI Formation, Short Circuit, Side Reactions, Unstable SEI, Volume Change

Candidate Failure Mechanisms:
Active Material Loss, Aging, Degradation, Dendrite Growth, Electrolyte Decomposition,
     Exothermic Reactions, Gas Evolution, Internal Short Circuit, Lithium Plating, Mechanical
      Stress, Overheating, Phase Transition, Polysulfide Shuttle, SEI Decomposition, SEI
     Growth, Side Reactions, Structural Degradation, Thermal Runaway, Transition Metal
     Dissolution, Volume Change

Based on the battery structure and observation data provided above, as well as the related
     historical cases, please infer the top-1 most likely Failure Causes and Failure
     Mechanisms of the system. Select exactly 1 items from each candidate list.

Answer in the following JSON format:
{
  "Failure Cause": ["...", "...", "..."],
  "Failure Mechanism": ["...", "...", "..."]
}
```

Generated Output:

Generated Output

```
{
  "Failure Cause": ["High Temperature"],
  "Failure Mechanism": ["SEI Growth"]
}
```

Based on the above case study results, this comparative analysis of a retired electric vehicle battery exhibiting capacity fade reveals the significant advantages of consulting historical cases over isolated analysis in identifying root causes and underlying physicochemical mechanisms.

The initial isolated analysis attributed the failure to ”Aging” and ”Active Material Loss.” While accurate at a macroscopic level in describing performance degradation, this conclusion has fundamental limitations in analytical depth. ”Aging” is an umbrella term that describes the phenomenon of performance degradation over time, rather than specifying the driving factors. Similarly, ”Active Material Loss” describes the outcome of reduced functional components in the electrode but fails to clarify the specific chemical or physical pathways involved.

In contrast, by integrating relevant historical case data, the analysis derived a more precise conclusion, identifying ”High Temperature” as the root cause and ”Solid Electrolyte Interphase (SEI) Growth” as the

failure mechanism, which aligns with the ground truth established in the original case. Historical case libraries (e.g., Cases 1, 2, 3, and 5) validate that "High Temperature" is a key stress factor accelerating degradation, while "SEI Growth" is the core electrochemical mechanism leading to irreversible consumption of recyclable lithium ions and subsequent capacity fade. This mechanism directly explains the primary pathway of "Active Material Loss."

Overall, this cognitive shift from macroscopic phenomena to microscopic mechanisms greatly enhances the actionability of the conclusion, as comprehensively compared in Table 10. Identifying the root cause as manageable "High Temperature" rather than the uncontrollable "Aging" provides clear direction for improving battery thermal management systems and optimizing operating conditions. At the same time, accurately identifying "SEI Growth" as the dominant mechanism offers critical theoretical support for enhancing long-term cycling stability through material modifications, such as developing more stable electrolyte systems. This dual-driven method, powered by both failure case knowledge and LLMs, establishes the essential foundation for accurate lifespan prediction, effective longevity extension strategies, and improved product reliability.

Table 10: Comparative Analysis: Why Case-Referenced Inference is Superior

Feature	Inference Without Case Reference	Inference With Case Reference	Advantage Analysis
Failure Cause	Aging	High Temperature	**More specific and actionable.** Aging is uncontrollable, while temperature can be managed and optimized via the Battery Management System, providing clear improvement direction.
Failure Mechanism	Active Material Loss	SEI Growth	**More fundamental and mechanistic.** Identifies the specific chemical process causing active material loss, directly linking to core battery materials science issues.
Link to Observation	Directly links to capacity fade	Links capacity fade via a clear chemical pathway (lithium consumption)	**More complete logical chain.** Establishes a complete sequence: "Condition $\rightarrow$ Chemical Process $\rightarrow$ Performance Fade".
Consistency with Historical Data	Consistent with general knowledge but lacks specificity	Highly consistent with findings across multiple cases (e.g., high temperature causes SEI growth leading to capacity fade)	**More reliable conclusion.** Based on broad experimental and research consensus, not isolated speculation from a single case.

4.11 Interpretability and Reliability Evaluation

To evaluate the interpretability and reliability of our retrieval-augmented approach, we conducted two complementary analyses: (1) a comparative assessment of explanation quality, and (2) a statistical analysis of prediction changes before and after incorporating retrieved cases.

Explanation Quality Comparison

We conducted a comparative analysis of explanation quality between the base model (DeepSeek-V3.2) and our RAG-enhanced model (BattFailScholar). Both models produced correct predictions on the selected samples, allowing us to focus purely on the quality of their explanations. A total of 32 samples where both models predicted correctly were identified and evaluated by a domain expert, who rated the explanations on a scale from 1 to 10 based on the following criteria:

- **9-10 (Expert Level):** Explanation deeply connects failure mechanism to specific electrochemical/mechanical processes; evidence is precise and directly from data/cases; reasoning is rigorous and insightful.
- **7-8 (Proficient):** Clear explanation with good connection to battery degradation; evidence supports the diagnosis well; logic is coherent.
- **5-6 (Adequate):** Basic explanation of the failure; evidence is relevant but could be more specific; logic is reasonable but shallow.

- **3-4 (Limited):** Explanation is vague or superficial; evidence is weak or loosely connected; reasoning lacks clarity.
- **1-2 (Poor):** Explanation is confusing or misleading despite correct label; evidence doesn't support the diagnosis; illogical reasoning.

As shown in Figure 11, BattFailScholar consistently achieved higher explanation quality scores compared to DeepSeek-V3.2. BattFailScholar obtained an average score of 7.91, corresponding to the Proficient level, while DeepSeek-V3.2 achieved an average of 6.28, placing it in the Adequate level. The majority of BattFailScholar's explanations scored 8, indicating consistently proficient reasoning with well-supported evidence. In contrast, DeepSeek-V3.2's scores were predominantly clustered at 6, reflecting adequate but shallow explanations with relevant yet generic evidence.

BattFailScholar demonstrated superior performance in 31 out of 32 cases (96.9%), with only one instance where DeepSeek-V3.2 scored higher. These results indicate that incorporating retrieval-augmented generation substantially enhances the quality of failure analysis explanations, elevating them from adequate to proficient levels by providing deeper electrochemical reasoning, more specific evidence, and better integration of historical case knowledge.

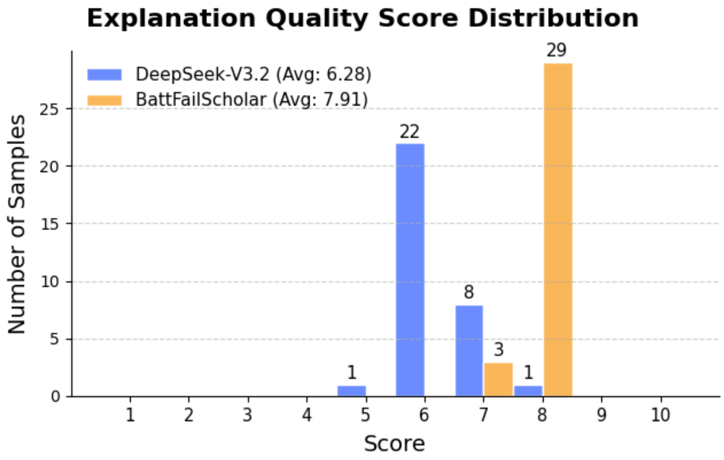

Figure 11: **Comparison of Generated Explanation Quality**

Prediction Change Analysis

To further assess the reliability of our approach, we analyzed prediction changes before and after incorporating retrieved cases. Figure 12 presents two complementary perspectives on model behavior.

The left subplot of Figure 12 shows that BattFailScholar consistently yields more corrections than new errors across all tasks, with net improvements of +36.0%, +18.3%, and +5.7% for Failure Cause, Failure Mechanism, and Detection Method respectively. The low rate of newly introduced errors (2.8%–5.8%) confirms the reliability of our approach.

The right subplot examines the proportion of corrected samples where the gold label exactly appeared in retrieved cases. The results show high ratios: **81.1%** for Failure Cause, **62.2%** for Failure Mechanism, and **73.3%** for Detection Method. This indicates that most successful corrections (average 72.2%) can be traced directly to exact matches in retrieved examples, providing transparent and verifiable reasoning paths. Notably, since this analysis is based on strict exact matching, the actual proportion of corrections supported by relevant context is likely higher when considering semantic variants.

Summary

These findings collectively demonstrate that BattFailScholar not only improves accuracy but does so in an interpretable and reliable manner. The explanation quality analysis shows that our approach produces significantly higher-quality explanations (7.91 vs. 6.28), elevating them from adequate to proficient levels.

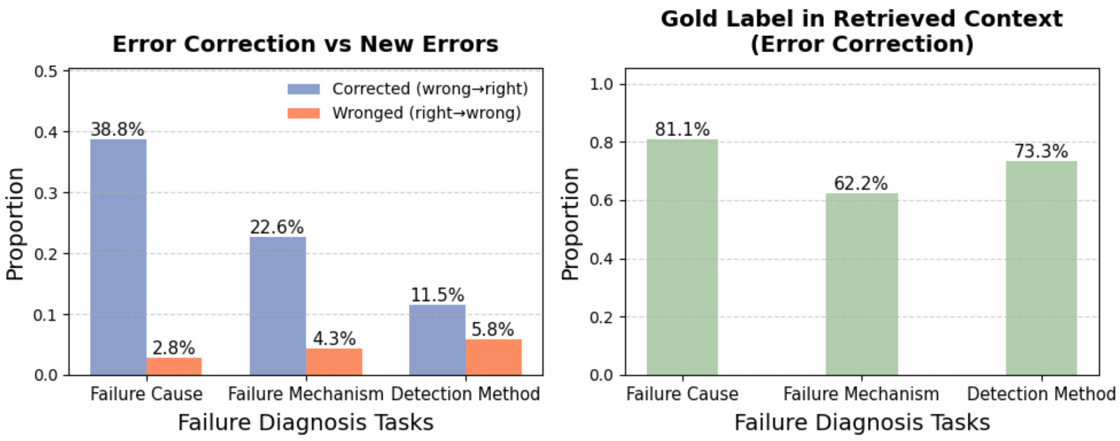

Figure 12: **Statistical Analysis of Prediction Changes with BattFailScholar**

The prediction change analysis confirms that most corrections (72.2%) can be traced directly to retrieved information, providing transparent reasoning paths, while the minimal introduction of new errors (2.8%–5.8%) further validates the reliability of our approach.

4.12 Efficiency Evaluation

In real-world diagnostic scenarios, response time directly impacts the efficiency of clinical workflows and user experience. Therefore, model inference efficiency is a key metric for evaluating the practicality of different approaches. To assess the inference efficiency of various models in diagnostic tasks, we compare the average processing time per query across different methods, including traditional deep learning models, LLMs, our proposed methods, and various GraphRAG variants. The results are summarized in Table 11.

Table 11: Comparison of Model Efficiency in Failure Diagnostic Tasks

**Category**	**Model / Method**	**Average Inference Time**
DL Models	MatBERT	3.29 ms
LLMs	DeepSeek-V3.2	3.31 s
	LLaMA3.1-8B	0.50 s
Our Methods	BattFailScholar (DeepSeek-V3.2)	3.77 s
	BattFailScholar (LLaMA3.1-8B)	1.73 s
	KNN + Our RRF	0.29 s
GraphRAG Variants	GraphRAG	37.1 s
	LightRAG	10.3 s
	SimGRAG	9.6 s
	T2RAG	30.2 s
	KG-Agent	15.8 s

For DL models, we include MatBERT[16] as a representative, which achieves an inference speed of 3.29 ms per query, demonstrating the efficiency advantage of lightweight fine-tuned encoder models.

For LLMs, in this evaluation, the API-based DeepSeek-V3.2[9] achieves an inference time of 3.31 s, which is primarily limited by network latency and server-side load of API calls. Alternatively, locally deployed models can be considered, such as the locally deployed LLaMA3.1-8B[12], which achieves an inference time of only 0.50 s, effectively avoiding the additional overhead of cloud-based services and providing more stable response times.

Our proposed BattFailScholar method introduces additional retrieval and verification mechanisms on top of base LLMs. The variants based on DeepSeek-V3.2 and LLaMA3.1-8B achieve inference times of

3.77 s and 1.73 s, respectively, representing increases of approximately 0.5 s and 1.2 s compared to their base models. This additional overhead is within an acceptable range. For scenarios with limited hardware resources or higher requirements for response speed, the KNN + Our RRF method can be considered. This method does not require local deployment of LLMs and achieves an extremely fast response time of 0.29 s with a slight reduction in performance.

Among GraphRAG variants[19, 20, 21, 22, 23], all methods require multiple requests to LLMs combined with knowledge graph interactions, resulting in generally high inference times, averaging above 10 seconds. These methods often involve long waiting times in practical use, making them difficult to satisfy the requirements of real-time diagnosis.

In summary, our proposed BattFailScholar method can be considered for scenarios seeking optimal performance within an acceptable time range, striking a good balance between performance and response time, particularly demonstrating clear advantages compared to other GraphRAG methods. For scenarios demanding extremely fast response times, BERT-based methods and the KNN + Our RRF method can be prioritized, achieving fast responses while maintaining reasonable performance.

4.13 Long-Tail Problem Experiment

To investigate the distribution characteristics of failure types in the constructed BF-KG, we conducted a statistical analysis of the sample counts across different failure causes and failure mechanisms. The results are presented in Figure 13 and Figure 14.

As shown in Figure 13, the failure cause types exhibit a pronounced long-tail distribution. Among the 15 failure cause categories, only 5 types (Overcharging, High Temperature, Electrolyte Decomposition, Volume Change, and High Current Density) have sample counts exceeding 100, accounting for 33.3% of the total categories but contributing 77.8% of the total samples. In contrast, the remaining 10 low-frequency types (including Low Temperature, Mechanical Stress, Short Circuit, and others) account for 66.7% of the categories but only 22.2% of the total samples. Some types, such as Side Reactions and Unstable SEI, have fewer than 20 samples, making reliable diagnosis extremely challenging.

Similarly, Figure 14 reveals a comparable long-tail pattern for failure mechanisms. Among the 20 failure mechanism categories, only 6 types (SEI Growth, Lithium Plating, Electrolyte Decomposition, Volume Change, Mechanical Stress, and Thermal Runaway) have sample counts exceeding 100, representing 30.0% of the categories but 73.5% of the total samples. The remaining 14 low-frequency types constitute 70.0% of the categories but only 26.5% of the total samples. Notably, many mechanisms such as Polysulfide Shuttle and Degradation have extremely limited samples (33 occurrences each), posing significant challenges for accurate diagnosis.

This pronounced long-tail distribution highlights a critical challenge in battery failure diagnosis: while numerous failure types exist in practice, the available case data is heavily skewed toward a few common types. For low-frequency failure types, traditional data-driven methods often suffer from insufficient training samples, leading to poor diagnostic reliability. This observation underscores the necessity of developing specialized methods that can effectively handle the long-tail problem. Our proposed drift-aware correction mechanism addresses this challenge by leveraging chemical system consistency to detect and correct erroneous predictions for low-frequency types, as detailed in Section 3.1.

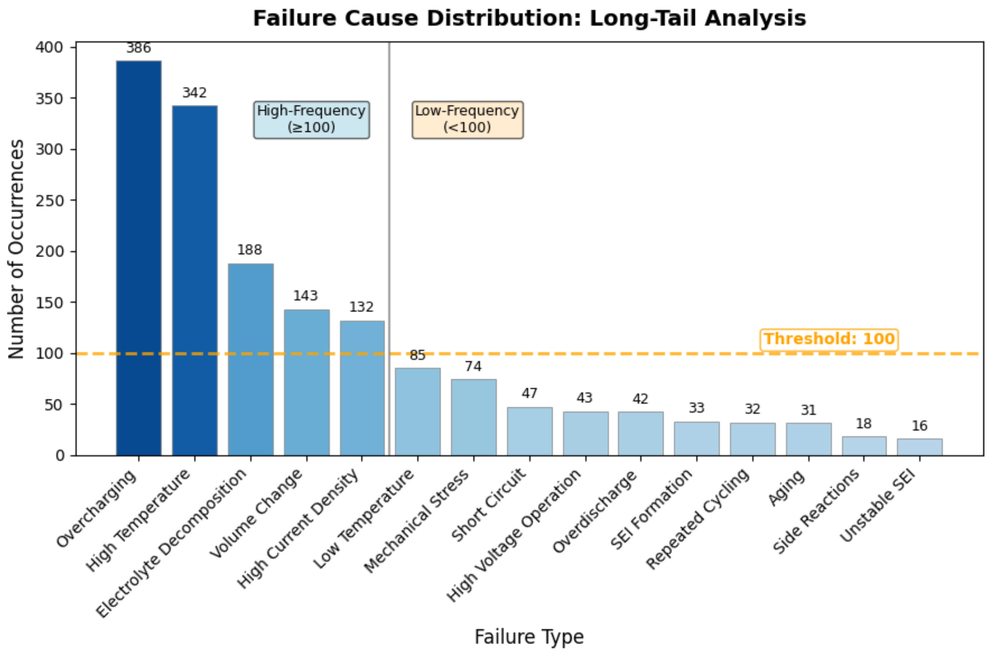

Figure 13: **Failure Cause Distribution: Long-Tail Analysis**

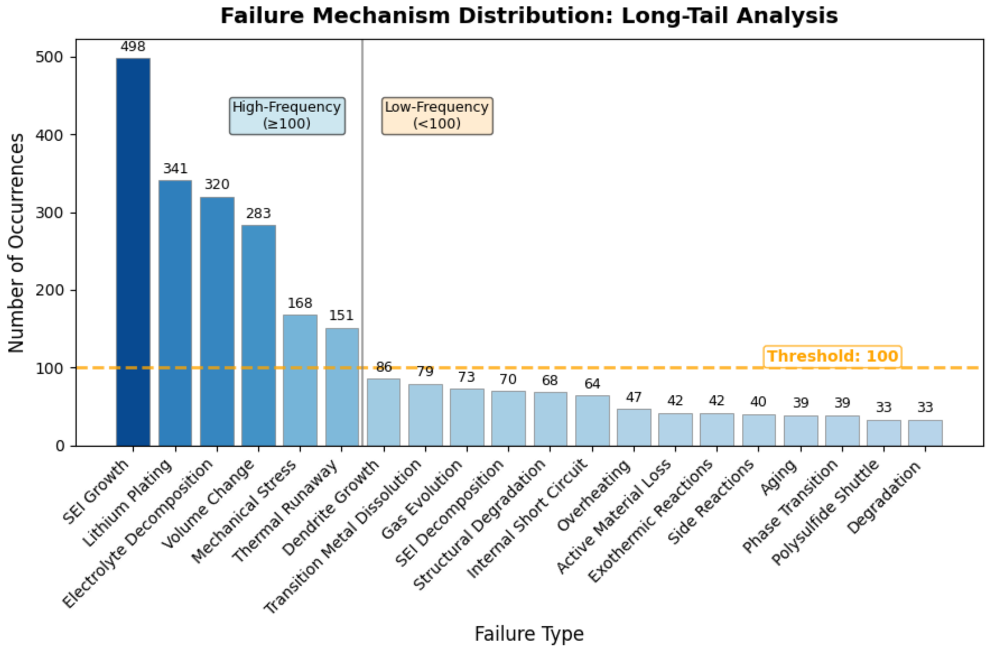

Figure 14: **Failure Mechanism Distribution: Long-Tail Analysis**

To further validate the effectiveness of the drift-aware correction mechanism, we analyzed the relationship between prediction change counts (number of changed labels within top-5 predictions) and diagnostic accuracy. The results are presented for high-frequency and low-frequency types across both failure cause and failure mechanism diagnosis.

Before Drift-Aware Correction

Table 12 shows the prediction change statistics before applying the drift-aware correction mechanism.

Table 12: Prediction Change Analysis (Before Correction)

Type	Change Count	Samples (%)	Base Acc (%)	KAG Acc (%)	Acc Change (%)
High-Frequency (Cause)	4	1.4	25.00	75.00	+50.00
	3	15.9	25.00	93.18	+68.18
	2	41.3	56.14	96.49	+40.35
	1	31.9	86.36	89.77	+3.41
	0	9.4	88.46	88.46	0.00
Low-Frequency (Cause)	4	2.0	100.00	0.00	-100.00
	3	15.7	62.50	50.00	-12.50
	2	41.2	57.14	61.90	+4.76
	1	27.5	85.71	92.86	+7.14
	0	13.7	100.00	100.00	0.00
High-Frequency (Mechanism)	4	3.7	80.00	90.00	+10.00
	3	21.5	84.48	94.83	+10.34
	2	37.8	87.25	95.10	+7.84
	1	26.7	94.44	95.83	+1.39
	0	10.4	100.00	100.00	0.00
Low-Frequency (Mechanism)	4	3.5	0.00	50.00	+50.00
	3	7.0	75.00	50.00	-25.00
	2	40.4	52.17	82.61	+30.43
	1	40.4	82.61	82.61	0.00
	0	8.8	80.00	80.00	0.00

For high-frequency types, prediction changes after KAG enhancement are predominantly beneficial. This is because sufficient relevant cases, combined with the proposed retrieval algorithm, effectively augment the LLM's knowledge and correct previously incorrect predictions. In contrast, for low-frequency types, no consistent improvement is observed as the change count increases, and significant performance degradation occurs (e.g., -100.00% and -12.50% for failure cause, -25.00% for failure mechanism). This is due to the scarcity of relevant cases, which introduces noise rather than useful evidence.

After Drift-Aware Correction

Table 13 compares the performance of low-frequency types before and after applying the drift-aware correction mechanism.

Table 13: Low-Frequency Type Performance Comparison

Type	Change Count	Before Correction (%)	After Correction (%)
Cause	4	-100.00	0.00
	3	-12.50	+20.00
	2	+4.76	+4.76
	1	+7.14	+7.14
	0	0.00	0.00
Mechanism	4	+50.00	+50.00
	3	-25.00	0.00
	2	+30.43	+30.43
	1	0.00	0.00
	0	0.00	0.00

After applying the drift-aware correction mechanism, the negative impacts on low-frequency types are effectively eliminated. The correction mechanism detects cases where noisy retrieved cases are likely to cause misclassification and reverts them to base LLM predictions. This effectively mitigates the issue of correct predictions being turned into errors after KAG enhancement, while preserving the gains from corrections that were originally wrong, thereby improving diagnostic reliability for low-frequency types while maintaining performance for high-frequency types. A comprehensive comparison of high-frequency and low-frequency performance before and after correction is presented in Figure 5c of the main text.

4.14 Risk Assessment Experiment

Reliable risk assessment is a critical component of battery failure diagnosis, as it directly informs maintenance decisions and safety interventions. Unlike classification-based diagnosis that merely identifies failure types, risk assessment provides actionable insights into the severity of the detected issues, enabling prioritization of responses in real-world applications.

Risk Level Annotation

To establish a benchmark for evaluating risk assessment capabilities, each test sample was manually annotated with one of three risk levels based on the severity of the failure scenario. The annotation followed the guidelines defined by domain experts:

- **L1 (Low Risk)**: Only performance-related issues, no safety concerns. Examples include gradual capacity fade due to normal aging, minor SEI growth, or reversible side reactions that do not compromise battery safety.
- **L2 (Medium Risk)**: Clear warning signs that could lead to safety issues if ignored. Examples include accelerated capacity loss, significant lithium plating with potential for dendrite formation, or electrolyte decomposition that may evolve into more severe conditions.
- **L3 (High Risk)**: Immediate safety hazards requiring urgent intervention. Examples include thermal runaway, internal short circuits, severe overcharging, or conditions with imminent fire or explosion risk.

Risk Level Distribution

The statistical distribution of risk levels across the 327 test samples is presented in Figure 15. Among all samples, L2 (moderate risk) constitutes the largest proportion with 160 samples (48.9%), followed by L3 (severe risk) with 103 samples (31.5%), and L1 (low risk) with 64 samples (19.6%).

This distribution reflects the practical reality of battery failure diagnosis: while moderate-risk scenarios are most common, a substantial proportion of cases involve immediate safety hazards that demand accurate identification. The presence of a significant number of high-risk cases underscores the critical importance of developing reliable risk assessment methods that can accurately distinguish between risk levels, particularly for L3 scenarios where misdiagnosis could lead to severe consequences.

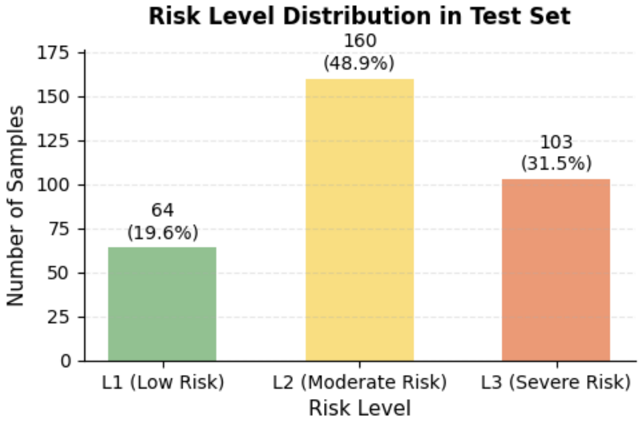

Figure 15: **Risk level distribution in the constructed risk assessment benchmark**

Failure-Risk Pattern Library Statistics

To support the proposed pattern matching-based voting method, a failure-risk pattern library was constructed by statistically analyzing correlations between failure factors (causes, mechanisms, forms) and their associated risk levels. The library contains patterns at three granularity levels: single-factor (individual cause,

mechanism, or form), two-factor (cause+mechanism, cause+form, or mechanism+form), and three-factor (complete failure chains). Only patterns with dominant risk level confidence $\geq$ 60% and minimum sample count $\geq$ 2 were retained.

Table 14 presents the scale of the constructed failure-risk pattern library.

Table 14: Failure-Risk Pattern Library Scale

**Pattern Type**	**L1**	**L2**	**L3**	**Total**
Single-factor	14	27	25	66
Two-factor	28	65	73	166
Three-factor	14	39	39	92
**Total**	**56**	**131**	**137**	**324**

The library comprises 324 patterns in total. Two-factor patterns constitute the largest proportion (166 patterns, 51.2%), followed by three-factor patterns (92 patterns, 28.4%) and single-factor patterns (66 patterns, 20.4%). Notably, L2 and L3 risk levels have substantially more patterns than L1 (L1: 56, L2: 131, L3: 137), reflecting the greater diversity of moderate- and high-risk failure scenarios.

Tables 15, 16, and 17 present the most frequent patterns for each risk level.

Table 15: Top-10 Representative Patterns for L1 (Low Risk)

**Rank**	**Pattern**	**Frequency**
1	[Form] Capacity Fade	63
2	[Mechanism] SEI Growth	55
3	[Mechanism+Form] SEI Growth → Capacity Fade	54
4	[Cause] High Temperature	40
5	[Cause+Form] High Temperature → Capacity Fade	40
6	[Cause+Mechanism] High Temperature → SEI Growth	39
7	[Full Chain] High Temperature → SEI Growth → Capacity Fade	39
8	[Cause] Electrolyte Decomposition	7
9	[Cause+Form] Electrolyte Decomposition → Capacity Fade	7
10	[Cause+Mechanism] Electrolyte Decomposition → SEI Growth	6

For L1 (low risk), the dominant patterns center on SEI growth and high temperature-induced capacity fade, which are recognized performance degradation mechanisms without immediate safety concerns.

Table 16: Top-10 Representative Patterns for L2 (Medium Risk)

**Rank**	**Pattern**	**Frequency**
1	[Form] Capacity Fade	139
2	[Mechanism] SEI Growth	102
3	[Mechanism+Form] SEI Growth → Capacity Fade	102
4	[Cause] High Temperature	96
5	[Cause+Form] High Temperature → Capacity Fade	90
6	[Cause+Mechanism] High Temperature → SEI Growth	80
7	[Full Chain] High Temperature → SEI Growth → Capacity Fade	80
8	[Cause] Electrolyte Decomposition	19
9	[Cause+Form] Electrolyte Decomposition → Capacity Fade	17
10	[Cause] Volume Change	16

For L2 (medium risk), while capacity fade and SEI growth remain dominant, the frequencies are substantially higher. The appearance of volume change as a warning signal aligns with the medium-risk characterization.

For L3 (high risk), the pattern composition shifts markedly. Overcharging and lithium plating become the dominant factors, replacing the SEI growth patterns seen in lower risk levels. Thermal runaway appears exclusively in L3, confirming the validity of the library's risk classification.

The failure-risk pattern library exhibits clear differentiation across risk levels: L1 is dominated by SEI growth and high temperature-induced degradation; L2 shows similar patterns with higher frequencies and additional warning signals such as volume change; L3 shows a marked shift to overcharging and

Table 17: Top-10 Representative Patterns for L3 (High Risk)

Rank	Pattern	Frequency
1	[Cause] Overcharging	58
2	[Form] Capacity Fade	56
3	[Mechanism] Lithium Plating	52
4	[Mechanism+Form] Lithium Plating → Capacity Fade	50
5	[Cause+Mechanism] Overcharging → Lithium Plating	45
6	[Cause+Form] Overcharging → Capacity Fade	45
7	[Full Chain] Overcharging → Lithium Plating → Capacity Fade	44
8	[Form] Thermal Runaway	32
9	[Cause] High Temperature	16
10	[Mechanism] Electrolyte Decomposition	12

lithium plating patterns, with thermal runaway uniquely present. This progressive pattern shift validates the rationality of the library construction and supports its use for interpretable risk assessment.

Advantages of Our Approach on Risk Assessment

Our pattern matching-based voting method offers two key advantages for risk assessment:

1. Superior Performance on High-Risk Cases: As shown in Figure 5f of the main text, our method achieves significantly improved accuracy for L3 (severe risk) predictions compared to conventional LLM-based approaches. This is particularly important given the safety-critical nature of high-risk scenarios.

2. Interpretable Risk Assessment: Unlike black-box LLM methods that produce confidence scores without justification, our pattern matching-based approach provides interpretable reasoning. Each risk prediction is supported by explicit evidence from the failure-risk pattern library.

This transparency enables domain experts to verify the reasoning behind risk predictions, facilitating trust and adoption in safety-critical applications. The supporting evidence can also be used for post-hoc analysis and continuous improvement of the pattern library.

Notably, the hybrid integration with LLM-based predictions enhances performance for high-risk cases while maintaining the interpretability advantages of the pattern-based method, offering a flexible solution for practical deployment.

Section 5. Battery Failure Chain Completion

5.1 Battery Failure Chain Statistics

This section presents statistical analysis of failure chains extracted from NMC battery failure cases. The analysis identifies 629 complete failure chains (Form→Mechanism→Cause), along with 427 partial chains missing failure causes and 161 partial chains missing failure mechanisms, indicating areas where diagnostic knowledge remains incomplete.

Summary Statistics:

- Complete failure chains: 629
- Failure Form → Mechanism chains (missing cause): 427
- Failure Form → Cause chains (missing mechanism): 161

Table 18: Complete Failure Chains (Top-10)

Failure Chain
Failure Form [Capacity Fade] → Failure Mechanism [SEI Growth] → Failure Cause [High Temperature]
Failure Form [Capacity Fade] → Failure Mechanism [Lithium Plating] → Failure Cause [Overcharging]
Failure Form [Swelling] → Failure Mechanism [Gas Evolution] → Failure Cause [Electrolyte Decomposition]
Failure Form [Capacity Fade] → Failure Mechanism [Lithium Plating] → Failure Cause [Low Temperature]
Failure Form [Capacity Fade] → Failure Mechanism [Cation Mixing] → Failure Cause [Ni2+ Migration]
Failure Form [Capacity Fade] → Failure Mechanism [Lithium Plating] → Failure Cause [Concentration Polarization]
Failure Form [Capacity Degradation] → Failure Mechanism [SEI Growth] → Failure Cause [High Temperature]
Failure Form [Thermal Runaway] → Failure Mechanism [Internal Short Circuit] → Failure Cause [High Temperature]
Failure Form [Capacity Fade] → Failure Mechanism [Lithium Plating] → Failure Cause [High Charging Current]
Failure Form [Capacity Fade] → Failure Mechanism [SEI Growth] → Failure Cause [Electrolyte Decomposition]

The distribution of complete failure chains is shown in Table 18, which presents the top 10 most frequent failure pathways. The most prevalent complete failure chain involves capacity fade driven by SEI growth under high temperature conditions, followed by lithium plating induced by overcharging. These patterns highlight thermal stress and charging protocols as critical factors in NMC battery degradation.

Table 19: Failure Form → Mechanism Chains (Missing Cause, Top-5)

Failure Chain
Failure Form [Capacity Fade] → Failure Mechanism [SEI Growth]
Failure Form [Capacity Fade] → Failure Mechanism [Lithium Plating]
Failure Form [Thermal Runaway] → Failure Mechanism [SEI Decomposition]
Failure Form [Capacity Fade] → Failure Mechanism [Electrolyte Decomposition]
Failure Form [SEI Growth] → Failure Mechanism [Electrolyte Decomposition]

For partial failure chains, Table 19 displays the top 5 Form→Mechanism chains where failure causes are missing. Partial chains lacking failure causes predominantly involve capacity fade mechanisms, with SEI

growth being the most common. This suggests established understanding of degradation mechanisms but incomplete attribution to root causes.

Table 20: Failure Form → Cause Chains (Missing Mechanism, Top-5)

**Failure Chain**
Failure Form [Thermal Runaway] → Failure Cause [Overcharging]
Failure Form [Thermal Runaway] → Failure Cause [High Temperature]
Failure Form [Capacity Fade] → Failure Cause [High Temperature]
Failure Form [Thermal Runaway] → Failure Cause [Short Circuit]
Failure Form [Thermal Runaway] → Failure Cause [Nail Penetration]

Similarly, Table 20 shows the top 5 Form→Cause chains where mechanistic explanations are missing. These chains are primarily associated with thermal runaway events, where overcharging and high temperature are identified as triggers but the detailed failure pathways require further investigation.

5.2 Failure Chain Completion Details

The failure chain completion task is designed to evaluate the model's ability to infer missing causal relationships in battery degradation pathways. Given partial failure information (e.g., observed failure forms with missing mechanisms or causes), the system must complete the causal chain by predicting the most plausible missing elements.

Data Construction

The test data for this task is constructed from the previously established failure diagnosis test set (Section 4.1) through a systematic masking procedure. Starting from the 327 FCFM test cases that contain complete failure chains (Failure Form, Failure Mechanism, and Failure Cause), two types of masking are applied to create incomplete chains:

- **Failure Mechanism masking**: The Failure Mechanism entity is removed from the chain, requiring the model to infer it from the available Failure Form and contextual information.
- **Failure Cause masking**: The Failure Cause entity is removed, requiring inference based on the Failure Form and Failure Mechanism.

Each test case retains all other information from the original case, including battery material specifications, experimental conditions, and observed failure symptoms. For focused analysis on specific chemistries, an NMC-based subset comprising 189 cases is used for detailed evaluation.

Evaluation Approaches

Three distinct approaches were implemented and compared for this task:

1. **Direct LLM inference**: The base LLM receives the incomplete failure chain along with battery context information, relying solely on its internal knowledge to predict the missing elements. No external references are provided.
2. **Rule-based statistical method**: A frequency-based approach that analyzes complete failure chains in the BF-KG to identify the most common completions for given partial patterns. For a case with missing Failure Cause, the method identifies all chains sharing the same Failure Form and Failure Mechanism, selecting the most frequent Failure Cause as the prediction. Similarly, for missing Failure Mechanism, the most common Mechanism associated with the observed Failure Form is selected.
3. **BattFailScholar (KAG)**: The proposed knowledge-augmented approach retrieves the most relevant historical cases from BF-KG based on the incomplete query, providing contextual evidence to guide the LLM's reasoning. The retrieval algorithm identifies cases with similar material properties, experimental conditions, and failure patterns, enabling evidence-based completion.

Implementation Details

For the BattFailScholar approach, the retrieval process follows the algorithm described in Section 2. For each test case:

- The incomplete case is converted to a structured query graph
- Top-K similar cases are retrieved from BF-KG using the proposed failure feature-aware retrieval algorithm
- Retrieved cases are provided as reference context in the prompt
- The LLM generates completions for the masked elements based on both the query context and retrieved evidence

For the rule-based method, the statistical patterns are derived from the complete failure chains in BF-KG. The frequency analysis considers:

- Chains matching the same Failure Form and Failure Mechanism (for Cause completion)
- Chains matching the same Failure Form (for Mechanism completion)
- Global frequency of Failure Causes and Mechanisms as fallback options

Prompt Configuration

For both the direct LLM and BattFailScholar approaches, the input is structured to present the incomplete failure chain clearly. The following example illustrates the prompt format for a case with missing Failure Cause:

```
Battery degradation analysis for failure chain completion:
Battery Information:  - Cathode Material:  NMC811 - Anode Material:  Graphite - Electrolyte:
1M LiPF6 in EC/DMC
Observed Symptoms:  - Failure Form:  Capacity Fade (detected after 589 cycles, 90% SOH
maintained) - Post-mortem finding:  Lithium plating observed on anode surface
Current Failure Chain:  Failure Form "Capacity Fade" --hasFailureMechanism--> Failure
Mechanism "Lithium Plating" Failure Mechanism "Lithium Plating" --hasFailureCause-->
[MISSING]
Task:  Based on the battery information and observed symptoms above, infer the most likely
Failure Cause that completes this degradation chain.
Candidate Failure Causes:  Low Temperature, High Current Density, Overcharging,
Overdischarge, Aging, SEI Formation, Electrolyte Decomposition, Mechanical Stress
Return your answer in JSON format:  "Failure Cause":  "..."
```

When retrieval is enabled for BattFailScholar, relevant historical cases with similar material compositions and failure patterns are inserted before the task instruction, providing concrete evidence to support the completion decision.

This task allows systematic comparison of each method's ability to reconstruct incomplete failure chains under controlled conditions. By evaluating performance on both mechanism and cause completion for NMC-based batteries, we can assess the potential of knowledge-augmented approaches to uncover previously undocumented failure pathways in real-world scenarios.

Section 6. Framework Application and Limitations

6.1 Diagnostic Platform and Application Example

Based on the proposed BattFailScholar framework, we have developed a diagnostic platform, as illustrated in Figure 16, to implement intelligent battery failure analysis in practical scenarios.

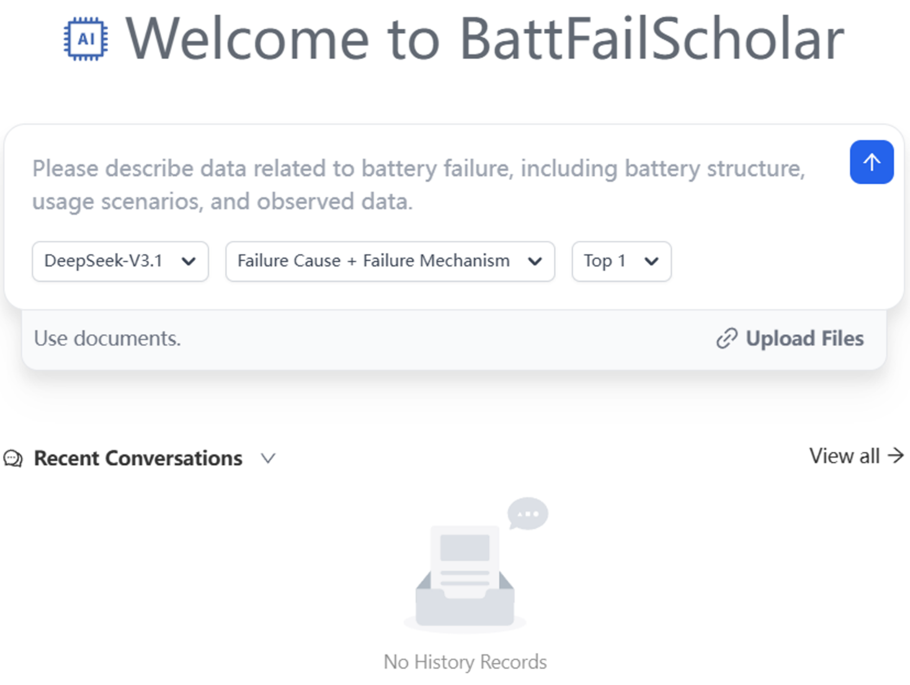

Figure 16: **Platform Interface.** The platform conducts failure analysis through a conversational interface, performing comprehensive failure diagnosis based on input data including battery structures, usage scenarios, and experimental data. As the proposed BattFailScholar method features a plug-and-play architecture, the platform supports the selection of various LLMs as the base model, tailored to specific analytical requirements such as identifying failure causes, elucidating underlying mechanisms, or recommending appropriate detection methods. Additionally, the system allows users to configure the number of candidate answers to be generated, with flexible options including Top 1, 3, or 5 responses.

The following example, as shown in Figures 17 to 19, illustrates the capabilities of the proposed BattFailScholar in diagnosing a typical battery failure case.

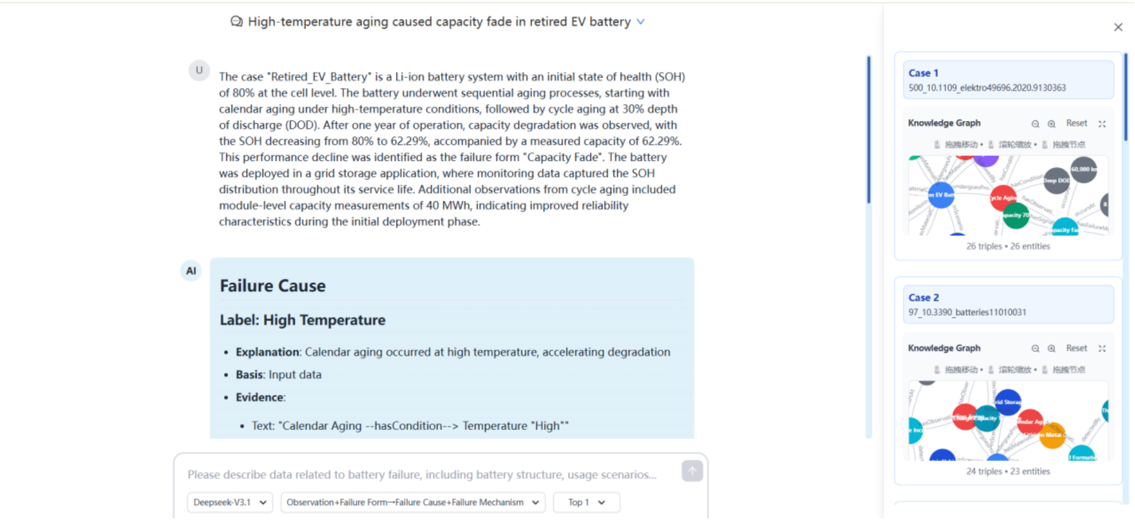

Figure 17: **Diagnostic Response Format**. Based on the input battery failure description and selected configuration settings, BattFailScholar automatically parses the query and employs the proposed FFR method to retrieve relevant reference cases from the BF-KG. These cases are provided to the LLM for context, enabling it to generate and output diagnostic results. Simultaneously, the referenced case graphs are displayed on the right-hand side for further consultation. This approach establishes a transparent and evidence-based diagnostic workflow, significantly enhancing the interpretability and reliability of battery failure analysis.

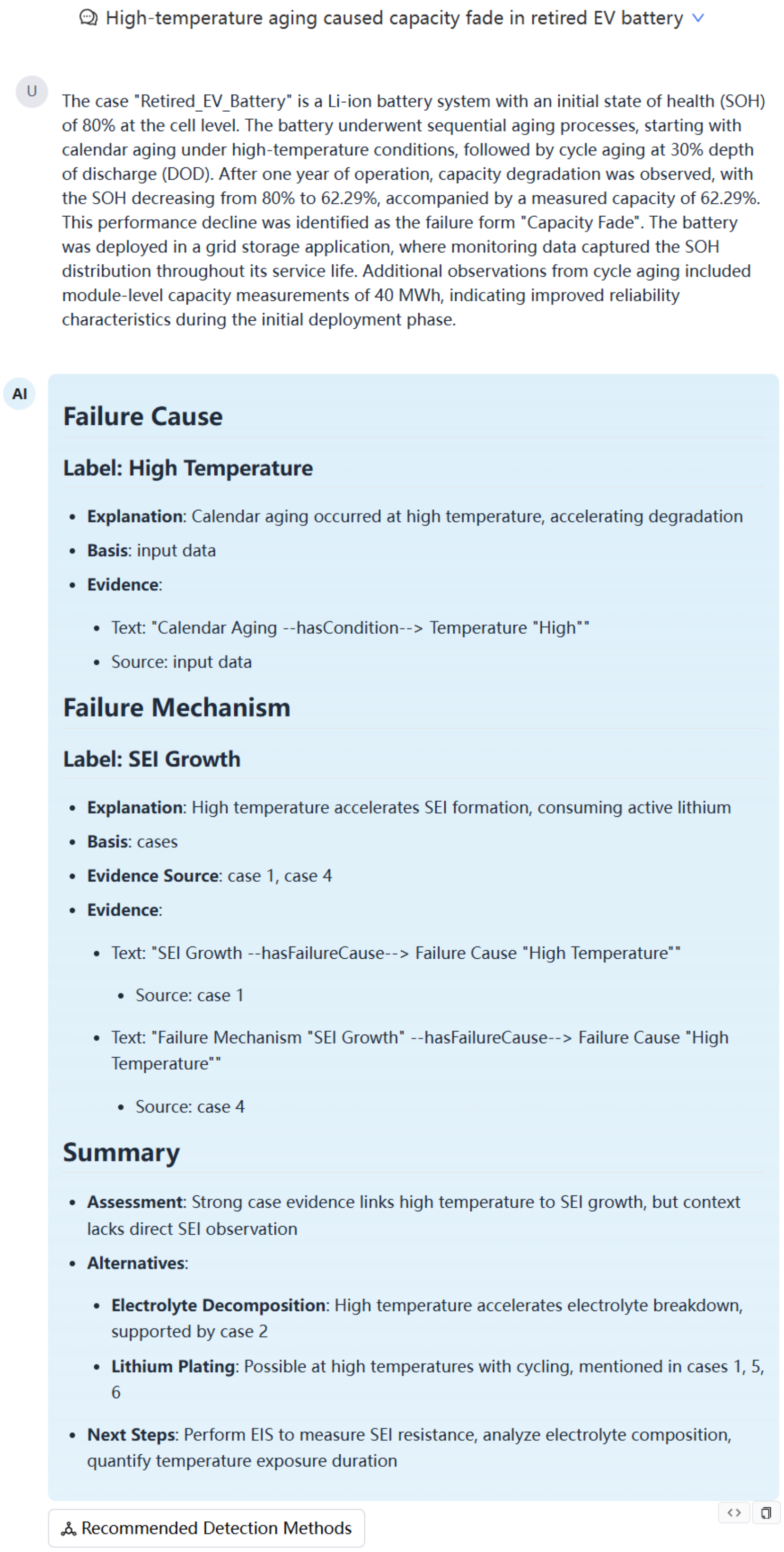

Figure 18: **Diagnostic Response Details**. In addition to the specific diagnostic results, the response includes relevant explanations, supporting evidence, source information (indicating which cases were referenced), and a comprehensive summary. It also presents potential alternative failure mechanisms and provides recommendations for next steps.

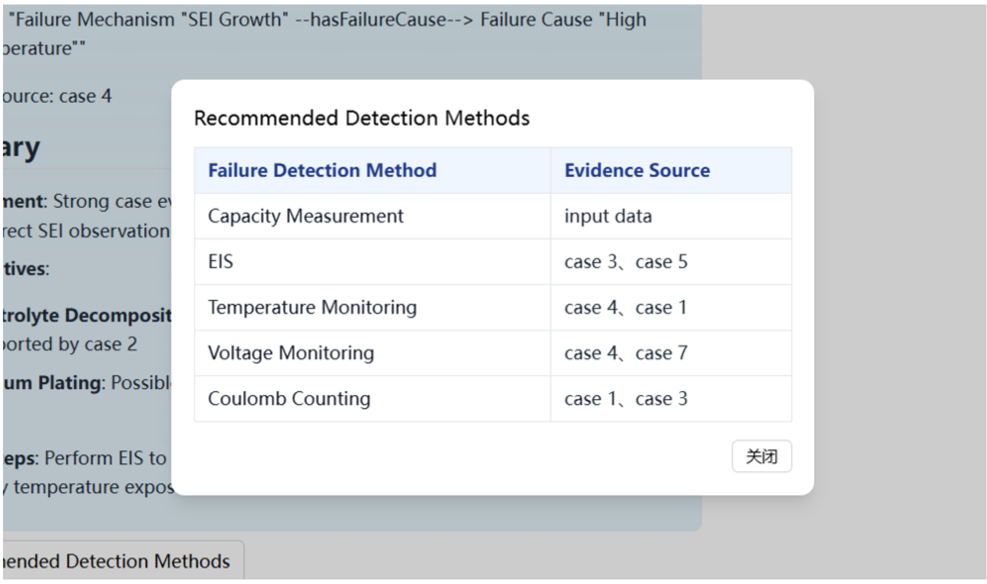

Figure 19: **Detection Method Recommendation.** Beyond diagnostic analysis, the platform leverages the retrieved relevant cases to infer scientifically validated detection methods, thereby establishing a knowledge-driven pathway from failure diagnosis to precise testing strategy formulation. This approach transforms traditional experience-based inspection into an evidence-based testing paradigm.

6.2 Limitations

Despite the promising diagnostic performance demonstrated by BattFailScholar, several limitations remain.

Data Comprehensiveness. The coverage of the knowledge graph relies on existing literature and may not promptly include emerging or rare failure modes. Although the drift-aware correction mechanism falls back to the base LLM's internal knowledge as an alternative, accurate identification of entirely unprecedented patterns cannot be guaranteed. However, this issue can be mitigated through continuous automated literature mining and graph updating mechanisms, which is an inherent advantage of retrieval-augmented approaches.

Multimodal Integration. The framework has not yet integrated multimodal information such as electrochemical impedance spectroscopy, thermal imaging, and gas sensing data, which often provide complementary diagnostic evidence in complex failure scenarios.

To address these issues, future work will explore fusion strategies for multimodal data to further enhance the framework's diagnostic capabilities.

References

[1] William Wheeler, Yann Bultel, Pascal Venet, and Ali Sari. An ageing study of twenty 18650 lithium-ion graphite/lfp cells in first and second life use. *Scientific Data*, 12(1):392, 2025.

[2] Jan Figgener, Jonas van Ouwerkerk, David Haberschusz, Jakob Bors, Philipp Woerner, Marc Mennekes, Felix Hildenbrand, Christopher Hecht, Kai-Philipp Kairies, Oliver Wessels, et al. Multi-year field measurements of home storage systems and their use in capacity estimation. *Nature Energy*, 9(11):1438–1447, 2024.

[3] Leo Wildfeuer, Alexander Karger, Deniz Aygül, Nikolaos Wassiliadis, Andreas Jossen, and Markus Lienkamp. Experimental degradation study of a commercial lithium-ion battery. *Journal of Power Sources*, 560:232498, 2023.

[4] Donal P Finegan, Julia Billman, Jacob Darst, Peter Hughes, Jesus Trillo, Matt Sharp, Alex Benson, Martin Pham, Inez Kesuma, Mark Buckwell, et al. The battery failure databank: Insights from an open-access database of thermal runaway behaviors of li-ion cells and a resource for benchmarking risks. *Journal of Power Sources*, 597:234106, 2024.

[5] Mina Naguib, Junran Chen, Phillip Kollmeyer, and Ali Emadi. Thermal fault detection of lithium-ion battery packs through an integrated physics and deep neural network based model. *Communications Engineering*, 4(1):1–9, 2025.

[6] Matthieu Dubarry, Nahuel Costa, and Dax Matthews. Data-driven direct diagnosis of li-ion batteries connected to photovoltaics. *Nature communications*, 14(1):3138, 2023.

[7] Yunwei Zhang, Qiaochu Tang, Yao Zhang, Jiabin Wang, Ulrich Stimming, and Alpha A Lee. Identifying degradation patterns of lithium ion batteries from impedance spectroscopy using machine learning. *Nature communications*, 11(1):1706, 2020.

[8] Gabriele Piombo, Simone Fasolato, Robert Heymer, Marc Hidalgo, Mona Faraji Niri, Simona Onori, and James Marco. Unveiling the performance impact of module level features on parallel-connected lithium-ion cells via explainable machine learning techniques on a full factorial design of experiments. *Journal of Energy Storage*, 84:110783, 2024.

[9] Aixin Liu, Aoxue Mei, Bangcai Lin, Bing Xue, Bingxuan Wang, Bingzheng Xu, Bochao Wu, Bowei Zhang, Chaofan Lin, Chen Dong, et al. Deepseek-v3.2: pushing the frontier of open large language models. arXiv: 2512.02556.

[10] Qwen. Qwen3-max: Just scale it. `https://qwen.ai/blog?id=241398b9cd6353de490b0f82806c7848c5d2777d`, 2025.

[11] OpenAI. Gpt-5 officially released. `https://openai.com/gpt-5`, 2025.

[12] Meta. Introducing llama 3.1: Our most capable models to date. `https://ai.meta.com/blog/meta-llama-3-1`, 2024.

[13] Zhiheng Huang, Wei Xu, and Kai Yu. Bidirectional lstm-crf models for sequence tagging. *arXiv preprint arXiv:1508.01991*, 2015.

[14] Jacob Devlin, Ming-Wei Chang, Kenton Lee, and Kristina Toutanova. Bert: Pre-training of deep bidirectional transformers for language understanding. In *Proceedings of the 2019 conference of the North American chapter of the association for computational linguistics: human language technologies, volume 1 (long and short papers)*, pages 4171–4186, 2019.

[15] Iz Beltagy, Kyle Lo, and Arman Cohan. Scibert: A pretrained language model for scientific text. In *Proceedings of the 2019 Conference on Empirical Methods in Natural Language Processing and the 9th International Joint Conference on Natural Language Processing (EMNLP-IJCNLP)*, pages 3615–3620, 2019.

[16] Leigh Weston, Vahe Tshitoyan, John Dagdelen, Olga Kononova, Amalie Trewartha, Kristin A Persson, Gerbrand Ceder, and Anubhav Jain. Named entity recognition and normalization applied to large-scale information extraction from the materials science literature. *J Chem Inf Model*, 59(9):3692–3702, 2019.

[17] Thomas N Kipf and Max Welling. Semi-supervised classification with graph convolutional networks. In *International Conference on Learning Representations (ICLR)*, 2017.

[18] Petar Veličković, Guillem Cucurull, Arantxa Casanova, Adriana Romero, Pietro Lio, and Yoshua Bengio. Graph attention networks. In *International Conference on Learning Representations (ICLR)*, 2018.

[19] Darren Edge, Ha Trinh, Newman Cheng, Joshua Bradley, Alex Chao, Apurva Mody, Steven Truitt, Dasha Metropolitansky, Robert Osazuwa Ness, and Jonathan Larson. From local to global: A graph rag approach to query-focused summarization. *arXiv preprint arXiv:2404.16130*, 2024.

[20] Zirui Guo, Lianghao Xia, Yanhua Yu, Tu Ao, and Chao Huang. Lightrag: Simple and fast retrieval-augmented generation. 2024.

[21] Yuzheng Cai, Zhenyue Guo, Yiwen Pei, Wanrui Bian, and Weiguo Zheng. Simgrag: Leveraging similar subgraphs for knowledge graphs driven retrieval-augmented generation. In *Findings of the Association for Computational Linguistics: ACL 2025*, pages 3139–3158, 2025.

[22] Shengbo Gong, Xianfeng Tang, Carl Yang, et al. Beyond chunks and graphs: Retrieval-augmented generation through triplet-driven thinking. *arXiv preprint arXiv:2508.02435*, 2025.

[23] Jinhao Jiang, Kun Zhou, Wayne Xin Zhao, Yang Song, Chen Zhu, Hengshu Zhu, and Ji-Rong Wen. Kg-agent: An efficient autonomous agent framework for complex reasoning over knowledge graph. In *Proceedings of the 63rd Annual Meeting of the Association for Computational Linguistics (Volume 1: Long Papers)*, pages 9505–9523, 2025.

[24] David Leake, Xiaomeng Ye, and David J Crandall. Supporting case-based reasoning with neural networks: An illustration for case adaptation. In *AAAI Spring Symposium: Combining Machine Learning with Knowledge Engineering*, volume 2, 2021.

[25] Jason Wei, Xuezhi Wang, Dale Schuurmans, Maarten Bosma, Brian Ichter, Fei Xia, Ed Chi, Quoc Le, and Denny Zhou. Chain-of-thought prompting elicits reasoning in large language models. In *Advances in Neural Information Processing Systems*, volume 35, pages 24824–24837, 2022.

[26] Donal P Finegan, Julia Billman, Jacob Darst, Peter Hughes, Jesus Trillo, Matt Sharp, Alex Benson, Martin Pham, Inez Kesuma, Mark Buckwell, et al. The battery failure databank: Insights from an open-access database of thermal runaway behaviors of li-ion cells and a resource for benchmarking risks. *Journal of Power Sources*, 597:234106, 2024.
